# Advanced materials for high-performance electrochemical water-splitting: a review of recent breakthroughs, and future prospects

**DOI:** 10.3389/fchem.2026.1829431

**Published:** 2026-05-08

**Authors:** Momna Qayyum, Sammia Shahid, Sana Mansoor, Urooj Fatima, Mohsin Javed, Salah Knani, Reem Alreshidi, Shahid Iqbal

**Affiliations:** 1 Department of Chemistry, School of Science, University of Management and Technology, Lahore, Pakistan; 2 Center for Scientific Research and Entrepreneurship, Northern Border University, Arar, Saudi Arabia; 3 Department of Physics, College of Science, Northern Border University, Arar, Saudi Arabia; 4 Department of Chemical and Environmental Engineering, University of Nottingham Ningbo China, Ningbo, China

**Keywords:** electrocatalysts, electrochemical water splitting, green energy, HER, non-noble metal-based catalyst, OER, sustainability

## Abstract

The sluggish HER/OER kinetics and the lack of dependable, highly efficient electrocatalysts limit the large-scale use of electrochemical water splitting, despite its potential as a sustainable hydrogen generating technique. This review presents comprehensive and mechanistically informed evaluation of the advanced electrocatalysts with particular emphasis on non-noble metal-based systems, including nanostructure surfaces, layer double hydroxides (LDH), metal-organic frameworks (MOFs), high-entropy materials (HEMs), perovskites, graphene-based materials, and covalent-organic frameworks (COFs). This review provides a unified framework for structure, property, and performance by correlating the strategies for the catalysts design, such the heteroatom doping, defect engineering, and the hybrid interface construction, with that of the key performance metrics including the current density, stability, cell voltage, and overpotential. Additionally, the influence of the operating conditions is also considered to offer more realistic perspective on the performance of the catalysts across the different electrochemical environments. Through the critical evaluation of the recent advancements, this review identifies key trends governing the catalytic behavior including the role of the active site engineering, interfacial effects, and the modulation of the electronic structure. This review outlines the actionable strategies which are aimed at bridging the gap between the laboratory-scale studies and the industrial water splitting, thus offering a rational framework for the development of the next-generation electrocatalysts for sustainable hydrogen production.

## Graphical Abstract

**Figure:**
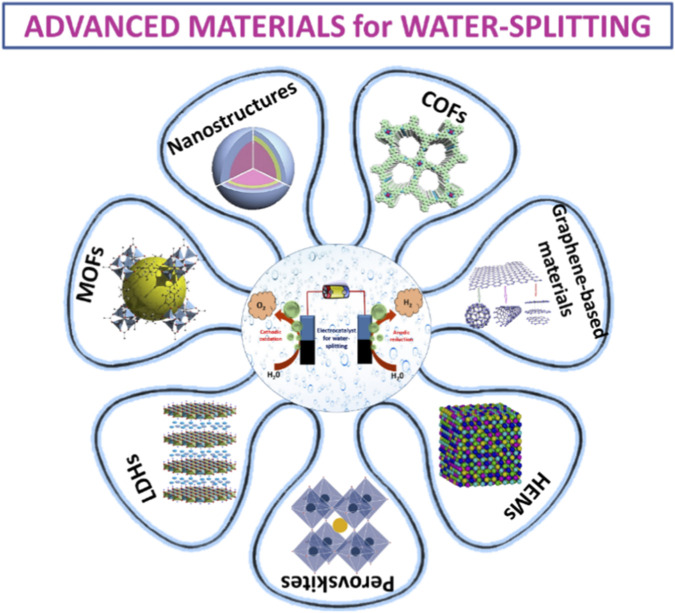

## 1 Introduction

Fossil fuels’ exhaustion is a warning sign for global warming, emphasizing the necessity to find affordable, more sustainable, and renewable energy sources (Selvanathan et al., 2024). Increased energy demands and ecological issues result from overpopulation, industry, and economic growth. Consequently, achieving carbon neutrality has emerged as a compelling global goal (Zhang X. et al., 2023). Other possibilities for addressing energy demands include renewable energy sources like solar, tidal, and wave power, but their availability is inconsistent due to regional and seasonal fluctuations (Chu and Majumdar, 2012). Due to its outstanding energy conversion efficiency, zero carbon dioxide emissions with only water as a byproduct, superior energy density (120 MJ kg^−1^) over gasoline (44 MJ kg^−1^) (Raveendran et al., 2023), and environmental friendliness, hydrogen is referred to as the fuel of the future (Yang J. et al., 2023).

Since no natural hydrogen is produced on Earth, hydrogen is generated by steam-reforming hydrocarbons at high pressure and temperatures, which inevitably leads to the consumption of reducing non-renewable energy bases and the atmospheric release of carbon dioxide (CO_2_) (Azizan et al., 2020). Additionally, the H_2_ produced by this process is supplemented by C, N, and S oxides, which would contaminate the catalyst’s surface and shorten its cycle life (Nogales-Delgado et al., 2023). Other techniques include using solar light energy to produce H_2_ in photoelectrochemical water splitting (Gopinath and Marimuthu, 2022). Their poor solar to hydrogen (STH) conversion efficiency imply that they create inadequate quantities per unit of time and cannot be used in place of bulk and instantaneous production, even if they are more ecologically friendly and produce pure hydrogen (Shwetharani et al., 2019). Reactive metals and metal hydrides can be hydrolyzed to produce large amounts of H_2_ efficiently (Ouyang et al., 2022). However, their precursors which are often hazardous metals are created by the fine chemical industries, which pollutes the environment and makes it impossible to use a greener manufacturing approach (Ouyang et al., 2022).

The water-splitting reaction is the most promising technique among the various energy conversion strategies designed to produce hydrogen gas as a clean chemical energy source (Niu et al., 2021). By transforming electrical energy from renewable resources into chemical energy and producing H_2_ through electrocatalytic generation, electrochemical water splitting is considered environmentally safe and a viable solution to the intermittent issue (Sahin et al., 2023). The electrochemical water-splitting that transforms water, the precursor, into hydrogen gas, is a promising strategy for future development because it produces hydrogen without causing pollution and offers sustainable regeneration and significant reactant preservation (Yu et al., 2021). A popular area of study among researchers is how to enhance the performance of water electrolyzers by creating inexpensive, high-performance water-splitting electrocatalysts (Xu et al., 2020).

The cathode, anode, and electrolyte are the three components of the electrolytic cell, which was first proposed in 1789 (Yan et al., 2016). The electrochemical splitting of water occurs *via* the following general reaction:

$$H_{2}O=H_{2\left(g\right)}+\frac{1}{2}\;O_{2\left(g\right)}$$ (1)

**FIGURE 1:**
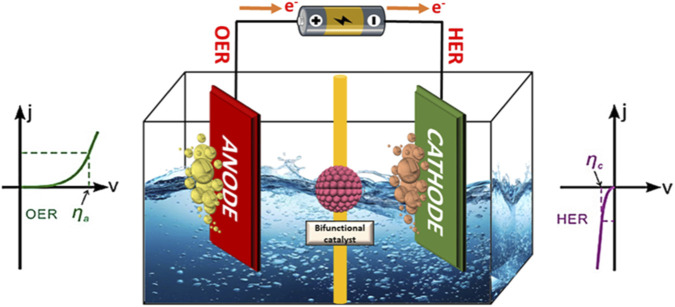
Schematic illustration of an electrocatalytic cell for water splitting.

Two half-electrode reactions (Equation 1) are involved in electrochemical water splitting (Zhu et al., 2019). As seen in Figure 1, OER occurs at the anode while HER is observed at the cathode (Farhan et al., 2023). These reactions are generally carried out in either acidic or alkaline solutions to guarantee effective ion transfer. It is observed that in both the alkaline and acidic media, the OER and HER processes are different (Cherevko et al., 2016), in the following manner:

In alkaline solutions:

$$Anode\;\left(OER\right):4{OH}^{-}\to O_{2\left(g\right)}+2H_{2}O+4e^{-},E^{\theta }(O_{2}/\;{OH}^{-}\;)=0.40\;V$$ (2)

$$Cathode\;\left(HER\right):4H_{2}O+4e^{-}\to 2H_{2\left(g\right)}+4{OH}^{-},E^{\theta }(H_{2}O/\;H_{2}\;)=-0.83\;V$$ (3)

$$Overall\;reaction:2H_{2}O\to 2H_{2\left(g\right)}+O_{2\left(g\right)},E^{\theta }=1.23\;V$$ (4)

In acidic solutions:

$$Anode\;\left(OER\right):2H_{2}O\to O_{2\left(g\right)}+4H^{+}+4e^{-},E^{\theta }(O_{2}/(H_{2}O)=1.23\;V$$ (5)

$$Cathode\;\left(HER\right):4H^{+}+4e^{-}\to 2H_{2\left(g\right)},E^{\theta }\left(H^{+}/H_{2}\;\right)=0\;V$$ (6)

$$Overall\;reaction:2H_{2}O\to 2H_{2\left(g\right)}+O_{2\left(g\right)},E^{\theta }=1.23\;V$$ (7)

According to Equations 2–7, water has a theoretical decomposition voltage of 1.23 V in both alkaline and acidic solutions, (Su et al., 2019). However, in practical applications, driving water splitting requires a higher applied voltage, because of additional significant overpotential that needs to be overcome, raising the expense of energy consumption (Xiao et al., 2022). Contact and solution resistance, the reaction’s slow kinetics, and the electrolyzer’s overpotential are the causes of the potential increase (Javed et al., 2023). As a result, pragmatic approaches for lowering the overpotential of the two half-reactions (HER and OER) integral to water-splitting must be developed.

To better understand these kinetic limitation, it is crucial to consider underlying reaction mechanism of the HER and OER. The HER proceed through the three fundamental steps: Volmer, Heyrovsky, and Tafel mechanisms, the kinetics of which are strongly influenced by that of the reaction environment (Sen et al., 2025). In electrocatalysis HER mechanism begins with the Volmer step (H* ↔ H+ + e^−^ + *), which involve the adsorption of the hydrogen intermediate (H*) onto the surface of the electrocatalyst, a process driven by the dissociation of water (in alkaline media) or proton reduction (in acidic media). This step is followed by the generation of the molecular hydrogen (H_2_) which is produced by one of the two pathways: Heyrovsky mechanism (H_2_ + * ↔ H* + H^+^ + e^−^), where the adsorbed hydrogen atom combines with that of an electrolyte proton and electron, or the Tafel process (H_2_ + 2* ↔ 2H*), where two adsorbed hydrogen atoms recombine to form H_2_ molecule (Gibson et al., 2026). While the Tafel process is significantly preferred in the catalyst with high density active sites, Heyrovsky process is preferred for catalysts with modest affinity for the H_2,_ where the rates of the adsorption and the desorption are well balanced. The reaction kinetics are very closely associated with that of the Gibbs free energy of hydrogen adsorption (ΔG_H*_) where values near the thermoneutrality (ΔG_H*=_≈ 0) facilitates the optimal catalytic performance (Xu et al., 2026). Notably the acidic environments facilitate the HER kinetics due to the availability of the proton, while alkaline media require additional water dissociation step, thus often resulting in the slower reaction rates.

**FIGURE 2:**
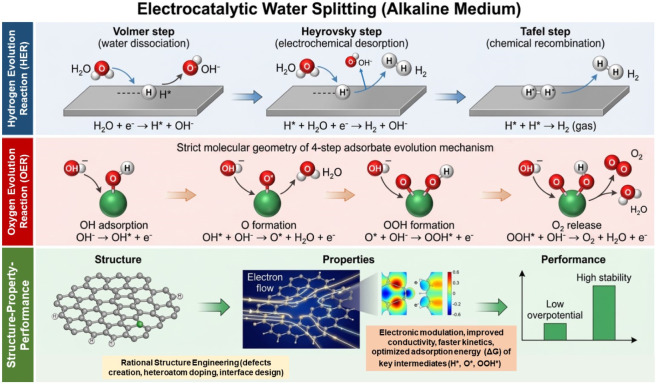
Schematic illustration of the HER and OER mechanism in the alkaline media, correlated with structure-property relationship governing the catalytic performance.

OER on the other hand, is more complicated four-electron (4e-) transfer process, that involves several surface-bound intermediates (Figure 2). It typically proceeds through the *OH, *O, and *OOH species before that of the oxygen evolution OER is typically the rate-limiting step in the overall water splitting owing to its slow kinetics which can be explained by large energy barriers associated to those of the intermediates (Li et al., 2025). The binding energies of reaction intermediates govern the OER activity; weakly bound -OH and strongly bound -O and -OOH determine poor kinetic activity, indicating difficult reaction initiation and slowed desorption. Similar to the HER, the electrolyte media affects the reaction pathway, however, OER is more favored in alkaline conditions because of the better stability and the reaction kinetics (Gebremariam et al., 2025). Therefore, overall water splitting heavily relies on the rational design of the electrocatalysts which must optimize the adsorption energetics, improve charge transfer, and maintain the structural stability in both of the acidic and alkaline media.

Although Ir- and Pt-based materials are frequently employed as benchmark electrocatalysts for hydrogen and oxygen evolution reactions, their large-scale applicability and widespread adaptation are hampered by their substantial cost, low abundance, and insufficient durability under extreme reaction conditions (Cai et al., 2022). Transition metal-based materials including, single-atom catalysts, metal oxides (TMO), (oxy)hydroxides (TMOH), metal alloys, and carbon-based materials, have drawn much interest as electrocatalysts for OER and HER but the use of non-precious metals in acidic environments is still problematic (Gebreslase et al., 2022).

Finding OER/HER electrocatalysts with large current density, minimal overpotential, and exceptional durability over time is imperative for commercial water splitting systems (Sun H. et al., 2023). Despite major advances in basic research merely 4% of hydrogen is generated annually by the electrocatalytic splitting of water, while 95% is produced through fossil fuel reforming annually (Lagadec and Grimaud, 2020). The distance between basic research and real-world applications hampers the expedited progress of water splitting. Testing procedures vary between laboratory and industrial settings, including extreme operating temperatures in highly corrosive electrolytes and appliances (Wang J. et al., 2020). Catalytic effectiveness assessments for engineered alkaline OER catalysts, for example, are typically conducted at room temperature with relatively small amounts of alkaline electrolytes, whereas commercial alkaline water electrolyzers operate at industrial temperature conditions with a highly corrosive electrolyte instead (Yu et al., 2022a).

In this study, the most advanced materials used for electrochemical-water splitting are reviewed. Here, we summarize the latest efforts to close the knowledge gap between basic science and practical electrochemical water splitting applications. Additionally, developments in catalyst designs for water splitting with industrial relevance are outlined, showing how material science and engineering are advancing practical applications and accelerating their synergies. In order to improve clarity regarding the application scenarios, it is very important to distinguish between the operating environment of the electrochemical water splitting. The systems are classified on the basis of both of the electrolyte medium and the nature of the ionic charge carriers. The acidic media (commonly H_2_SO_4_) are employed in the proton exchange membrane (PEM) electrolyzers, where the proton (H^+^) transport occur through the membrane, requiring highly stable and corrosion resistant electrocatalyts (Zhou et al., 2025). Whereas alkaline media (typically KOH) are associated with that of the anion exchange membrane systems (AEM) that employs a solid polymer membrane allowing zero-gap configuration where the hydroxide ions (OH^−^) act as the primary charge carriers. Although it currently face challenges with membrane stability and the ionic conductivity, it intends to combine the low-cost benefits of alkaline media with the high-performance dynamics of PEM (Muhyuddin et al., 2025). The conventional alkaline (ALK) rely on the liquid phase ion transport and are widely employed for the large-scale hydrogen production (Szabó et al., 2026). Owing to the fact that the catalysts performance is strongly dependent on the reaction media, all of the materials discussed in this review are categorized based on their electrolyte conditions. This classification offers a more realistic assessment of their suitability for the practical electrochemical technologies.

The unique contribution of this review which distinguishes itself from the existing literature lies in moving beyond the conventional material-wise categorization and establishing a unified framework that connects the catalyst structure, performance metrics, and the real-world application conditions. This review offers a data-driven evaluation of electrocatalysts’ performance across the diverse material classes, by systematically integrating the mechanistic insight with the experimentally reported parameters such as the current density, durability, cell voltage, and the overpotential. Ultimately, future perspectives of electrocatalyst design strategies are presented in an attempt to close the gaps between lab-scale research and large-scale industrial electrocatalysis operations. Thus, this integrated perspective provides the actionable insights for the fundamental material design of the scalable and high-performance electrocatalyts for hydrogen production.

## 2 Advanced materials for electrochemical water-splitting

Much work has been done to rationalize the relatively inexpensive electrocatalyst performances (Yu et al., 2018). Strategies for increasing electrocatalyst activity can be placed into two general categories. One is to use structural regulation to raise the apparent activity of the entire electrode. The other involves using compositional engineering to increase each active site’s intrinsic activity (Zhan et al., 2018). Recent advances in basic scientific research have yielded significant breakthroughs in the engineering of remarkable OER and HER electrocatalysts, many of which even fulfill the demands of industry. Some of these are described below:

### 2.1 Nanostructured surfaces

**FIGURE 3:**
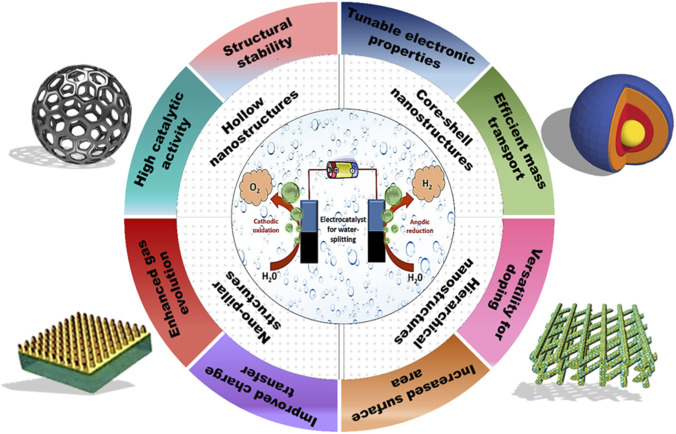
Key attributes of nanostructured surfaces as efficient electrocatalysts.

Nanostructured surfaces are defined as substrates with typical characteristics that range in size from 1 to 100 nm (although a maximum dimension of 100 nm can sometimes be relaxed to greater lengths, for the material and the property under examination). The recent spike in interest in these systems is due to the extraordinary outcomes that could arise from a significant size reduction. A system composed of a relatively small number of atoms transforms from a nearly infinite (and periodic) solid crystal, displaying intriguing improved characteristics (catalytic, electronic, magnetic, thermal, ferromagnetic, mechanical, optical, and selectivity) (Rosei, 2004). The use of nanostructured electrocatalyst materials in water splitting has shown promise as an affordable and environmentally friendly method of producing hydrogen. Figure 3 depicts the unique properties of nanostructure surfaces that contribute to their efficiency as catalysts for water-splitting. By changing the size and shape of the nanomaterial electrocatalysts, their properties can be adjusted. Some of the examples of nanostructured surfaces as potential electrocatalysts for water-splitting are listed below:

#### 2.1.1 Hollow nanostructures

Because of their many benefits, hollow nanostructures especially hollow heterostructured nanomaterials can offer a variety of ways to speed up the HER/OER kinetics thus making them an attractive option for commercial utilization (Tian et al., 2023). More specifically, there are plenty of easily available active sites due to the significant surface area of hollow nanostructures (Li and Zeng, 2019). In comparison to other high surface area nanostructured electrocatalysts, the hollow configurations exhibit potent confinement effects. More precisely, there is a noticeable improvement in the ability to prevent particle migration and aggregation in hollow-structured electrocatalysts with porous shells (Wang Z. et al., 2020). More importantly, controlling the shell structure could enable tunable mass transport during reactions (Hou et al., 2021). The optimal binding and desorption of reaction intermediates are made possible by the chemical compositions of hollow materials that can be adjusted. Moreover, hollow nanostructures can significantly decrease the reliance on costly precious metals (Yang M. et al., 2022). The utilization of hollow nanostructures to produce hydrogen *via* electrocatalysis has the potential to enhance catalyst apparent activity while decreasing the amount of noble Pt metal used. Schaak and colleagues have reported the synthesis of nickel phosphide (Ni_2_P) hollow spheres for HER through thermal decomposition (Popczun et al., 2013).

Hollow-structured cobalt-based electrocatalysts are considered to be potential electrocatalytic nanomaterials due to their low cost, large surface-to-volume ratio, plenty of active sites, tunable elemental composition, and minimal toxicity (Do et al., 2024). Chang et al. created CoP/MnO hollow nanofibers with a wider contact interface in order to maximize the OER catalytic functionality of transition metal phosphides (TMPs). Following 5,000 voltammetry measurement cycles, the composite material maintains its 263.5 mV overpotential. It has remarkable reliability for large-scale electrocatalysis, requiring only 230 mV at a current density of 10 mA cm^−2^ (Chang et al., 2022). For the electrocatalytic hydrogen evolution reaction (HER) to be commercialized on a large scale, stable and effective catalyst development is essential. Ru-Cu(OH)x/CF, an amorphous hollow copper hydroxide nanowire structure doped with Ru, was synthesized by Shen et al. Under a range of conditions, the electrocatalyst demonstrated remarkable long-term stability and impressive performance in driving 10 mA cm^−2^. To investigate high-performance catalysts and straightforward synthesis for HER, this approach might be useful (Shen et al., 2022). Since hollow nanostructures are widely used, they present a promising option for water-splitting electrocatalysts because of their excellent catalytic activity, structural stability, abundance of active sites, and tunable morphology (Yang L. et al., 2022).

#### 2.1.2 Core-shell nanostructures

Over the past few years, notable advancements have been achieved through the construction of core-shell nanostructures devoid of or containing minimal noble metals. The HER, OER, and overall water-splitting kinetics and electrocatalytic performance can be improved by utilizing their special properties, which include an extensively exposed reactive surface, tailored electronic configurations, strain-modulated effects, interface-driven synergy, or reinforced stability (Yin et al., 2020). To date, a wide range of inorganics (components related to transition metals and carbon) have been added to core-shell electrocatalysts. The highly exposed active surface of the core-shell functional materials can modify the charge distribution or chemical arrangement of the interfacial regions to promote an extended lifespan of corrosive electrolytes and create synergy between multiple functional sites on the core and shell (Fan et al., 2021).

A two-step method was used to prepare hybrid, core-shell Co/CoNx@C nanoparticles enveloped by Co-N doped carbon nanosheets. The Co-N-PC catalyst exhibits a half-wave potential of 0.833 V and a limiting current density of 5.70 mA cm^−2^, surpassing Pt/C catalysts, highlighting its superior electrocatalytic activity for the oxygen reduction reaction in alkaline media. It also shows greater methanol tolerance and electrochemical durability (Song et al., 2018). The potential of CoS_2_ as an electrocatalyst for the hydrogen evolution reaction (HER) was predicted theoretically by Jiang et al. A novel Ti_3_CNCl_2_@CoS_2_ core-shell nanostructure was created by combining CoS_2_ with Cl-terminated MXenes-Ti_3_CNCl_2_, which improved HER activity and offered a solid foundation for future design (Jiang et al., 2022). CuPd/Pd core/shell nanoparticles with an ultrathin Pd shell were created by Xie et al. These nanoparticles demonstrated Pt-like bifunctional reactivity for the oxygen reduction and hydrogen evolution reactions in acid electrolytes. The reaction kinetics were promoted by the compressive strain-induced downshift, which also increased the HER activity. The ideal CuPd/Pd core/shell NPs are among the best-reported Pd-based catalysts, with an overpotential of for 76 mV for HER, which is comparable to Pt (Xie et al., 2021).

#### 2.1.3 Nanoporous surfaces

High efficiency, low cost, and environmental friendliness are provided by electrochemical water splitting for hydrogen storage; however, pH universality, superior activity, durability, and low cost require sophisticated electrocatalysts (Xu et al., 2017). Extensive research has been conducted in the field of electrochemical water splitting on dealloyed nanoporous electrocatalysts with exceptional synergistic potential for active sites, significant intrinsic catalytic efficiency, substantial specific surface area, and distinctive mass and electron transport attributes (Qiao et al., 2023). The distinctive surface structure of nano-metals at the microscopic scale, combined with the bulk properties of metals at the macro level, makes nanoporous metals an excellent example of structural design (Liu et al., 2016). Specifically, electron conduction and mass transportation are supported by their bicontinuous structural characteristics, which include considerable accessible surface, metallic conduction networks, and a multitude of channels (Hu et al., 2022).

An advanced bifunctional electrode for HER and OER electrocatalysis has been reported by Shen et al. utilizing Ni foam with heteroatom-modified nanoporous Ni surfaces. The electrode has an optimized Gibbs free energy for the OER and retains metallic conductivity after being prepared using a Ni(OH)_2_-decorated NF. The Ni/Ni-N_0.28_/NF electrode outperforms Pt/C and RuO_2_ catalysts in terms of performance and durability, requiring a low overpotential of only 63 mV for the HER and 320 mV for OER (Shen et al., 2020). A nanoporous NiMnFeMo alloy was created by Liu et al. for water splitting in basic solutions. The three-dimensional nanoporous electrode in a basic electrolyte exhibits exceptional catalytic activity for hydrogen evolution and oxygen evolution reactions, even at high current densities (η_10_ = 1.54V for full water splitting) (Liu H. et al., 2021). Zeng et al. showed that in contrast to commercial RuO_2_ and other catalysts, FeCo/CeO_2_−xNx laminate nanoporous composites exhibit superior oxygen-evolution electrocatalysis in 1 mM KOH at ultra-low Tafel slope (∼33 mV dec^−1^) and a minimal overpotential of 360 mV. This makes them appealing candidates for large-scale hydrogen generation (Zeng et al., 2023).

#### 2.1.4 Hierarchical nanostructures

One-dimensional (1D) nanowires or nanotubes, two-dimensional (2D) nanosheets, and zero-dimensional (0D) nanoparticles are examples of low-dimensional or nanoscale sub-units that are typically arranged in an ordered fashion to form an integrated architecture known as a hierarchical nanostructure (Ko and Grigoropoulos, 2015). For an electrocatalyst to have a high mass activity, the material must be small enough to enable an enhanced surface-to-volume ratio, which will enable more active regions to be exposed to the electrolyte and take part in the reaction. But concurrently with the performance improvement, this size reduction also has unfavorable effects, like higher charge transfer resistance (R_ct_). In this instance, by ensuring smooth contact between the subunits, the catalyst can be fabricated into hierarchical nanostructures, which effectively avoids these issues (Fang et al., 2017). In addition, the use of hierarchical nanostructures can provide a number of other benefits, including increased electrolyte penetration, faster bubble release by creating more free space, and prevention of irregular nanoparticle agglomeration. Several investigations have demonstrated that using hierarchical nanostructures as electrocatalysts for water-splitting offers significant advantages (Chaudhari et al., 2017). In essence, all of the most active nonprecious electrocatalysts for HER, OER, and overall water splitting (OWS) that have been documented so far possess hierarchical structures (Fang et al., 2017).

Li et al. examined the impact of phosphorus concentration on the shape of CoP/C and its ability to catalyze water splitting in an alkaline environment. Even after 24 h, the hierarchical structure continues to show excellent activity and stability. Moreover, the hierarchical structure CoP/C used as the anode and cathode for water splitting achieves 10 mA cm^−2^ with only 1.56 V, providing a straightforward water-splitting approach (Li et al., 2020). Similarly, Singh et al. demonstrated a novel approach to the design of a bifunctional hierarchical nanostructure catalyst that exhibits excellent reversibility, low cost, high activity, and binder-free characteristics. With a small cell voltage of approximately 1.56 V at 10 mA cm^−2^, the 3D hierarchical O–Ni_1−x_W_x_Se_2_ on conductive nickel foam substrate (NF) exhibits outstanding robustness and exceptional catalytic performance for hydrogen evolution reaction (HER) and oxygen evolution reactions (OER) (Singh et al., 2022). In order to produce hydrogen through electrochemical water splitting, non-precious three hierarchical nanostructures of Ni_3_S_2_ on three-dimensional nickel foam, nanorods, multiconnected nanorods, and nanosheets were synthesized. The nanosheets yielded the best results; they needed an overpotential of 110 mV and 211 mV for HER and OER, respectively. The study offers information on how to enhance electrocatalytic activity and the environment for additional catalytic uses (Zubaid et al., 2024). Zhao et al. constructed a hierarchical Ni-CoFe_2_O_4_ electrode on nickel foam (NF) with a nanocone-nanoneedle design, enhances oxygen evolution reaction (OER) kinetics by creating a strong localized electric field that accelerates electrolyte ion accumulation (Zhao et al., 2026). Its high‐curvature nanostructure aids quick gas bubble detachment, reducing active site blockage, and allows stable performance at high current densities. Incorporating Co an electronic promoter improves interfacial electron transfer, resulting in a cell voltage of 1.75 V at 1.0 A cm^−2^ in an anion exchange membrane (AEM) water electrolyzer, maintaining stability for over 220 h. This work showcases the significant benefits of structural and electronic engineering in developing efficient OER electrocatalysts with improved mass transport.

#### 2.1.5 Nanopillars

A particular kind of nanomaterial structure called a nanopillar is made up of vertical columns with a diameter of several hundred nanometers. Low charge recombination is a feature of this morphology (Esmaili et al., 2021). Nanopillars are nanoscale surface modulations with a high aspect ratio that have a wide range of uses (Lin et al., 2023). With their shorter diffusion distance, nanopillars offer a structure that facilitates charge transport to the electrolyte interface and enhances light absorption through scattering (Samuel et al., 2020). Furthermore, when combined with materials of the proper physical and compositional properties, the substantial surface area and reduced density promote lightning-fast reactions and prevent recombination (Kim et al., 2018).

To create nickel nanopillar-array electrodes for alkaline electrochemical water splitting, nickel was electroplated into the pores of an anodic aluminum oxide (AAO) template. Increased threshold voltage and production efficiency were achieved by the nanopillars’ filling of the pores and strong adhesion to the conducting layer (Cheng et al., 2014). A hierarchical nanostructure was created using a sequential synthetic approach, where each component was set up to reach its maximum potential. To construct a massive surface area electrocatalyst with outstanding OER catalytic ability, robust structural features, electron transfer pathways, and excellent durability after 60,000 s of polarization, cobalt oxide (Co_3_O_4_) nanopillars were engineered onto ultrathin 1T-molybdenum sulfide (1T-MoS_2_) (Ying et al., 2022). Liu et al. created an ultra-hydrophilic and super-aerophobic structure by precipitating dense ternary NiMoFe alloy nanoparticles on hierarchical MoO_2_ nano-pillar arrays. These advanced dual-functioning electrodes deliver industrially advantageous current densities and retain an essentially constant potential over 1,000 h of water splitting. They also exhibit exceptional charge and mass transit capacity, a large number of active sites, and outstanding OER and HER activity (Riaz et al., 2022). Although the surface area and active site exposure are considerably increased by the nanostructuring strategies, compositional tuning can further improve the catalytic activity and stability. In this context, layered double hydroxides (LDHs) have emerges as an attractive class of materials owing to its inherent structural and physiochemical feature, tunable metal composition, as well as favorable electrical characteristics (Teli et al., 2025).

### 2.2 Layered double hydroxide (LDH)-based materials

**FIGURE 4:**
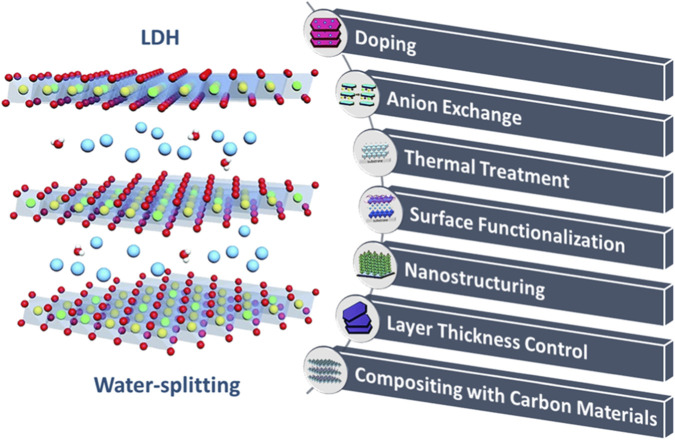
Strategies for modifying the characteristics of LDH-based materials.

LDHs are a class of two-dimensional materials characterized by a layered structure, that are related to hydrotalcite-like compounds or anionic clays (Riaz et al., 2024). Lamellar ionic materials, or LDHs, are composed of positively charged trivalent layers, compensating organic and inorganic species, and negatively charged water molecules. The layered cluster structure of LDHs is characterized by the placement of oxygen anions at the eight corners and transition metals (TM) at the center of each octahedron, represented as MO_6_ (Mishra et al., 2018). LDHs provide exceptional controllability, enabling modifications to their chemical and physical characteristics, such as water content, interlayer anion type, and metal ion proportion. They facilitate the creation of functionalized LDHs due to their structural adaptability and simplicity of surface modification (Li L. et al., 2024). Some of the techniques for adjusting the characteristics of LDH-based materials are demonstrated in Figure 4.

Layered double hydroxides (LDHs) and their derivatives, including oxy-hydroxides, oxides, carbides, sulfides, nitrides, selenides, and phosphides, have been extensively studied in recent years for their potential as bifunctional electrocatalysts. (Kulkarni et al., 2023). These derivatives offer many advantages over typical precious metals, including affordability, exceptional catalytic efficiency, robustness, and availability of resources (Yu et al., 2020a). Due to the presence of active hydroxyl groups and oxo-bridges, its surface exhibits intriguing qualities such as swelling traits, biocompatibility, substantial thermochemical durability, and flexible compositional tenability (Khan et al., 2023). Carbon/LDHs, cobalt–manganese (CoMn-LDHs), cobalt–iron (CoFe-LDHs), iron/aluminum (FeAl-LDHs), and nickel/iron (NiFe-LDHs) based layered double hydroxides are among the LDH-based catalysts that have shown superior performance for energy storage and energy transformation applications (Feng et al., 2017). Due to their adaptable and variable interlayer anions, LDH materials (such as NiFe-, NiCo-, CoFe-hydroxides, *etc.*) have emerged as one of the most guaranteeing abundant earth-transition metals-based electrocatalysts (Gao et al., 2021; Peng et al., 2022).

Two-dimensional (2D) materials based on Ni, Co, and Fe, as well as layered double hydroxides (LDH), are the most common non-precious-metal-based materials used in water splitting. These materials are inexpensive, have excellent electrocatalytic efficiency, and are easy to prepare. They demonstrate significant promise as a replacement for materials based on precious metals (Li et al., 2023). The highly active nickel-iron layered double hydroxide (NiFe LDH) catalyst is considered a promising candidate in the field of electrocatalysis. It demonstrates a very high turnover frequency and mass activity, which indicates its potential in the future hydrogen economy. This catalyst addresses practical challenges, such as achieving high efficiency and long durability at low overpotentials (Bodhankar et al., 2021). Iron in NiFe catalysts stabilizes important intermediates in HER, such as OH_ad_ on Fe^3+^ sites and H_ad_ on Ni^2+^ sites, encouraging a synergistic mechanism that speeds up the production of hydrogen. When compared to platinum, this self-optimizing ability significantly lowers overpotentials, and the Fe-O-Fe links stabilize key intermediates, reducing energy requirements (Xue et al., 2020).

In LDH-based electrocatalysts, the metal cations act as the active centers for OER where the reaction proceeds through the sequential adsorption of the intermediates. In the OER, the critical stage is the formation of O–O bonds by combining oxygen atoms, facilitated by water molecules or hydroxyl ions attacking metal-bound oxygen on a catalyst surface. Creating oxygen vacancies near metal–oxygen bonds reduces electron density, making these sites electrophilic and active for H_2_O or OH^−^ attack. This presence of oxygen vacancies also enhances electrical conductivity and accelerates charge transfer at the catalyst–electrolyte interface, improving the efficiency of the process (Zhu Y. et al., 2026). By electrochemically transforming CoFe Prussian blue nanocubes on nickel foam (NF) and doping with molybdenum, Zhao et al. were able to synthesize (Mo-doped CoFe LDH/NF) nanosheets successfully. Because of its powerful electron absorption capacity and abundance of edge defects, the catalyst demonstrated exceptional performance in overall water-splitting. In alkaline media, OER and HER needed a low overpotential in order to attain a current density of 100 mA cm^−2^. When utilized as a bifunctional electrode, it demonstrated long-lasting water-splitting durability in an alkaline electrolyte and a low potential of 1.55 V. The work enabled the rational design of bifunctional electrocatalysts with potential industrial applications (Zhao et al., 2022).

Yu et al. synthesized NiFeLa-LDH/v-MXene/NF that demonstrated good durability and decreased overpotentials for HER and OER. In comparison to the Pt/C-RuO_2_ couple, the alkaline electrolyzer powered by NiFeLa-LDH/v-MXene/NF produces a lower cell voltage (Yu et al., 2022b). In addition, hollow CuO@CoZn-LDH/CF nanoarray catalysts show good activity as bifunctional catalysts for both HER and OER in alkaline media with ultralow overpotentials. In comparison to commercial IrO_2_@CF||Pt/C@CF couple catalysts, it also exhibits an exceptionally low cell voltage alkali-electrolyzer (Yin et al., 2021). A hierarchical NiFe LDH/N-doped Co/nickel foam (NF) electrode was recently created by Wang et al. using a top-down building approach for anode design. This electrode was created using a hydrothermal-gas phase nitridation–electrodeposition technique (Wang J. et al., 2025). NiFe LDH nanoplates on N-doped Co nanowires supported by nickel foam substrates are included in this electrode. At an overpotential of 262 mV, it generates an impressive current density of 100 mA·cm^−2^ with little attenuation after 100 h. An anion exchange membrane water electrolyzer (AEMWE) system outperforms the majority of known catalysts, requiring just 1.63 V to achieve a current density of 1 Acm^−2^. By boosting surface area and strengthening catalyst–support contacts, the N-doped Co nanowires improve stability and activity. Using top-down oxidation etching of amorphous NiFeMo oxide in an alkaline environment, Guo et al. developed a scalable synthesis technique for Mo-modified NiFe LDH nanoflowers (Guo et al., 2025). The selective leaching of Mo results in a phase transition to an LDH structure that incorporates MoO_4_^2−^, which enhances the formation of catalytically active NiOOH during the OER. Maintaining etching products in slurry improves nanoparticle dispersion, yielding a highly conductive catalyst layer. The NFM-OED electrocatalyst shows exceptional OER performance with an overpotential of 260 mV at 10 mA cm^−2^, without needing conductive carbon additives. Additionally, a membrane electrode assembly with the NFM-OED anode operates at a cell voltage of 1.73 V at 1.0 A cm^−2^ in 1 M KOH at 60 °C, maintaining stability for 600 h at 1.0 A cm^−2^ and 100 h at 2 A cm^−2.^ These findings highlight the significance of intercalation modification of LDH catalysts and the need for catalyst preservation to optimize OER efficiency in AEM water splitting. Table 1 represents the performance metrics of some high-durability LDH-based electrocatalysts.

**TABLE 1:** Summary of the electrochemical properties of LDH-based materials for electrochemical water-splitting in alkaline media.

Catalyst	Electrolyte medium	Overall cell voltage(V)	Current density (mAcm^−2^)	Durability	References
NiFe-LDH@NiMoH2@NF	1 M KOH	1.61	500	1,000 h	Zhang et al. (2024a)
NiCoP@FeNi LDH	1 M KOH	1.70	1,000	30 h	Yang et al. (2023b)
Ni_3_Se_2_@NiFe-LDH/NF	1 M KOH	1.55	10	25 h	Hu et al. (2021)
NiFe-LDH/Ni/NM	1 M KOH	1.80	100	154 h	Zhou et al. (2022)
NiFe-LDH/NF-3.5	1 M KOH	1.86	500	230 h	Huang et al. (2022)
Co@NC-CNTs@NiFe-LDH	1 M KOH	1.66	10	760 h	Yan et al. (2022)
Ni/NiFe-LDH/IF	6 M KOH	1.87	1,000	1,500 h	Liao et al. (2023a)
CoP@NiFe LDH/Ni	1 M KOH	2.13	1,000	100 h	Liu et al. (2024a)
NiFe LDH@Ni_3_N/NF	1 M KOH	1.80	500	100 h	Wang et al. (2020c)
Ru_x_SACs@FeCo-LDH	1 M KOH	1.52	1,000	1,000 h	Mu et al. (2022)
c-CoMP/a-CoM LDH/NF	1 M KOH	1.722	500	85 h (@100 mAcm^−2^)	Zheng et al. (2022)
CoNiN@NiFe LDH	1 M KOH	1.63	10	100 h	Wang et al. (2021)
Ru-Ni_3_S_2_-NiFe LDHs/NF	1 M KOH	1.85	500	240 h	Wu et al. (2022)
FeCo-LDH/NM	1 M KOH	1.82	1,000	150 h	Sun et al. (2021a)
NiFe LDH/NiS	30% KOH	2.01	8,000	80 h	Wen et al. (2021)
H-CMS_x_@NiFe LDH/NF	1 M KOH	1.99	400	50 h	Lee and Park (2024)
NiFe LDH-D1	1 M KOH	1.94	100	72 h (@ 200 mAcm^−2^)	Zhang et al. (2023b)
Pt@S–NiFe LDH	1 M KOH	1.62	100	200 h	Lei et al. (2023)
FeNi-LDH@Ni	6 M KOH	1.74	10	100 h	Jia et al. (2023)
Ni_3_S_2_/Cu–NiCo LDH	1 M KOH	1.75	100	12 h	Jia et al. (2021)

The comprehensive assessment of the LDH-based electrocatalysts that are summarized in Table 1, demonstrate their exceptional suitability for the high current density electrochemical water splitting, particularly in the alkaline conditions. While the 1 M KOH serve as the standard benchmark for the electrochemical testing activity. The studies conducted at 6 M KOH was likely tested to simulate the high-power industrial conditions and evaluate the durability of the material under the accelerated alkaline stress. Most of the systems are constructed as self-supported hierarchical architectures on the conductive substrates (e.g., Ni foam, Ni mesh) which significantly enhanced charge transport and structural stability is observed. Interestingly, the reported cell voltages for these materials generally fall within the ranges of 1.5–1.9 V even at the elevated current densities of the 500–1,000 mAcm^−2^, highlighting their potential for the industrial-scale applications. Notably the system such as the NiFe LDH/NiS achieve an ultra-high current density up to the 8,000 mAcm^−2^. Even though at the expense of the increased cell voltage and reduced stability which indicates a trade-off between activity and that of the durability. In the context of the long-term and sustained performance some of the LDH-based systems demonstrated remarkable operational robustness for up to 1,500 h. This enhanced stability could primarily be attributed to *in situ* surface reconstruction of LDHs into the catalytically active metal (oxy)hydroxides phases, which serves as the true active sites during the OER. Moreover, the electronic structure can be modulated by the incorporation of the secondary phases (such as phosphides, nitrides, sulphides, single-atom catalysts) and the bimetallic synergy (Co-Fe, Ni-Fe, *etc.*), which can lead to the optimization of adsorption energetics and thus improving the reaction kinetics. Even though these observations establish LDH-based systems as one of the most practically viable catalytic platforms, additional efforts are required to extend their applicability beyond the alkaline conditions.

Despite their many advantages, LDH-based systems still face challenges related to the electronic conductivity and structural tunability, which limit precise control over the active sites necessitating alternative approaches. With their ordered porous structure, precise metal linker geometries, and adaptable coordination environments, metal-organic frameworks (MOFs) presents novel possibilities for the catalyst design (Tucker-Quiñónez et al., 2025).

### 2.3 Metal-organic frameworks (MOFs)

MOFs have emerged as significant materials in electrochemical water-splitting due to their unique properties, including periodic porosity, stable structures, exceptional channel qualities, high electrical conductivity, substantial specific surface area, and abundant active sites. (Nemiwal et al., 2021a). Their programmable structures facilitate the incorporation of various functional groups on their surfaces (Lu et al., 2022). While pristine and composite MOFs may exhibit low conductivity and instability in alkaline and acidic environments, their derivatives demonstrate improved stability. Following multiple heating treatments, MOFs become effective precursors for fabricating electrically active metal nanoparticles and other porous materials, showcasing exceptional catalytic performance, particularly for oxygen reduction and water splitting (Radwan et al., 2021). Characterized by their predictable chemical behavior and designable frameworks consisting of organic linkers and metal nodes serving as active sites, MOFs can be modified to include additional catalytic species (Wei et al., 2020). These modifications can include metals, porphyrins (Younis et al., 2020), ligands based on bipyridine phthalocyanines, and phosphines (Wei et al., 2020). With excellent permeability, MOFs can also be adapted to create conductive and electrochemically active forms suitable for HER or supported on conductive substrates, including carbon-based materials (such as CNTs, graphene, rGO), metallic foams, and metallic oxides, metal nitrides, and metal phosphides (Sowbakkiyavathi et al., 2025) through hybrid MOF development for enhanced performance (Su et al., 2023). Thus MOF, offers a well-defined coordination environment where the organic linkers influence the charge distribution and electronic structure whereas the metal nodes serve as the active sites. Their tunable porosity facilitates the mass transport and upon the hybridization or the partial transformation, enhanced conductivity enables efficient transfer of electrons for the HER and OER processes.

Using FeS_2_@MoS_2_ layers and 2-dimensional MOF-derived mesoporous CoS_2_ nanoarrays, a strong electrocatalyst for alkaline water splitting was created. With low cell voltages and exceptional stability over 30 h, this MOF-based nanostructure demonstrated superior activity in the overall water-splitting (OWS) (Chhetri et al., 2022). A novel method utilizing pulsed laser ablation in DMF (dimethylformamide) was employed to create 3D transition-metal-based MOF materials with different architectures, such as Cu–BTC, Ni–BTC, and Co–BTC. The materials were analyzed in terms of their integration, crystalline composition, phase purity, structure, thermal endurance, and oxidation states. The substance demonstrated strong activity and a low overpotential in electrocatalytic reactions, suggesting the possibility of developing novel bifunctional electrocatalysts with noble metal-free MOF materials (Shreyanka et al., 2022).

According to recent reports, NiCo(nf)-P, 2D MOF-derived NiCoP nanoflakes, is an extremely effective bifunctional electrocatalyst for both oxygen and hydrogen evolution reactions. With exceptional long-term stability over 30 h, it achieves 100/500/1,000 mA cm^–2^ and shows low overpotentials in 1.0 M KOH electrolyte solution for HER and OER at low cell voltages (1.74/1.86/1.94 V respectively) (Xu Z. et al., 2022). A practical technique for producing hydrogen on a large scale for industrial use is an anion exchange membrane water electrolyzer (AEM) that makes use of the alkaline hydrogen evolution reaction (HER). However, it faces challenges in maintaining consistent and tolerable operation at low overpotentials and ampere-level current densities as an HER catalyst. To address this, ultrafine Fe_2_P and Co_2_P nanoparticles are synthesized on N and P dually doped porous carbon nanosheets using a universal ligand-exchange modulation approach, resulting in a current density of 1,000 mA cm^−2^ (Zhang H. et al., 2023). Tran et al. described a synthetic procedure for creating a multi-metallic MOF-assembled hierarchical electrocatalyst. This electrocatalyst is extremely robust and active, making it suitable for reliable industrial-scale current density electrocatalytic splitting of seawater. Compared to Pt/C, the RhCoNi-MOF displays an exceptionally low overpotential of about 40 mV at a current density of 10 mA cm^–2^. The combination of Co and Rh metal ions has a synergistic effect that is responsible for its outstanding electrocatalytic effectiveness (Tran et al., 2024). Chu et al. reported a method to create a heterostructured electrocatalyst using Co(OH)_2_ nanosheets and *in situ* generated bimetallic MOF (NiFe-MIL) with ferronickel foam as the metal supply and conductive substrate (Chu et al., 2025). With a low overpotential of 230 mV at 10 mA cm^−2^, a minimal Tafel slope of 12.79 mV dec^−1^, and exceptional long-term stability of 1,000 h in alkaline environment, the Co(OH)_2_@NiFe-MIL/NFF catalyst demonstrates excellent OER electrocatalytic performance. This performance is explained by the synergistic effects of its distinctive interfacial structure and variety of active sites, suggesting a novel approach to electrocatalyst creation through electronic structure manipulation.

Diyali et al. demonstrated an operando electro-oxidation technique to produce cobalt oxyhydroxide (NBU-4/NF@CoOOH), a smart electrocatalyst made from a 2D cobalt(II) MOF built on nickel foam (NF) (Diyali et al., 2025). With Faradaic efficiencies of 97.1% and 93.4%, respectively, in 1 M KOH, this electrocatalyst reaches a remarkable overpotential of 76 mV for HER and 336 mV for OER at a current density of 10 mA/cm^2^. With a low voltage need of 1.65 V and outstanding stability over 12 h at a constant current, it exhibits bifunctionality for total water splitting. Its distinctive 2D hexagonal shape and microporous structure with zigzag NF flow channels that improve kinetics through dual site synergism of Co^3+^ and Ni^2+^ are attributable for the outstanding performance. In the quest for energy sustainability, the development of affordable *de novo* electrocatalysts *via* operando electro-oxidation techniques holds enormous potential for the scalable generation of green energy. Jiang et al. synthesized a MOF-derived 3-D Fe-doped CoF_2_ nanocubes on MXene (Fe-CoF_2_/MXene/NF) using a four-step method which comprise of electrochemical deposition, co-precipitation, ligand exchange, and *in situ* fluorination (Jiang et al., 2025). When compared to other transition metal fluorides, the improved catalyst performs better with low overpotentials of 121 mV for the HER and 210 mV for the OER at 10 mA cm^−2^. After 100 h of continuous operation, it maintains 98% activity while achieving successful total water splitting at a cell voltage of 1.52 V in 1 M KOH. Therefore, through logical structural design that integrates organic framework derivatization, metal doping, and interfacial engineering, this investigation presented a synergistic strategy to boost catalytic activity by concurrently modulating electronic configurations and accelerating charge transfer kinetics. The electrocatalytic performance of some MOFs is listed in Table 2.

**TABLE 2:** Comparative performance of MOF electrocatalysts in terms of overpotential, Tafel slope, cell voltage, and durability at high current densities.

Catalyst	Overpotential (mV)		Tafel slope (mV dec^−1^)		Overall cell voltage(V)	Current density (mAcm^−2^)	Durability	References
	OER	HER	OER	HER				
FeNi(BDC) (DMF,F)/NF	252	—	37.4	—	1.90	400 (1 M KOH)	30 h	Lin et al. (2019)
NFN-MOF/NF	360	293	58.8	35.2	∼1.80	500 (1 M KOH)	30 h	Senthil Raja et al. (2018)
NiFe(dobpdc)	251	170	36	69	—	100 (1 M KOH)	30 h (@10 mAcm^−2^)	Qi et al. (2020)
Er0.4 Fe-MOF/NF	326	—	73	—	—	1,000 (1 M KOH)	100 h (@100 mAcm^−2^)	Ma et al. (2021)
FCN-MOF/NF	284	—	29.5	—	∼1.541	1,000 (1 M KOH)	50 h	Raja et al. (2020)
MFN-MOFs/NF	294	234	55.4	30.1	1.80	500 (1 M KOH)	100 h	Raja et al. (2019)
Zn@Ni-MOF/NF	630	—	—	—	∼1.86	2000 (1 M KOH)	48 h (@100 mAcm^−2^)	Shrestha et al. (2023)
Ru-modified NiFe-MOF nanosheet (Ru-NiFe-③/NF)	310	90	86.0	42.7	1.47	100 (1 M KOH)	20 h (@50 mAcm^−2^)	Wang et al. (2022a)
IF@CoFe-TDPAT NSA	240	234	30.8	30.1	1.78	500 (1 M KOH)	100 h (@300 mAcm^−2^)	Hu et al. (2024)
MIL53 (NiFe)@NF	210	62	42	49	1.6	500 (1 M KOH)	18 h	Zhao et al. (2020a)
CoNC@P-MoS_2_	518	354	66	36	1.92	1,000 (1 M KOH)	—	Chang et al. (2024)
NCF/Ni-BDC	450	350	35.1	149.5	—	50 (1 M KOH)	45 h	Sadeghi et al. (2022)
NiFe-MS/MOF@NF	230	156	32	82	1.74	50 (1 M KOH)	28 h	Zhao et al. (2020b)
S-NiBDC	—	310	—	75	—	1,000 (1 M KOH)	150 h	Cheng et al. (2022)
NiV-MOF NAs	290	—	76.2	—	—	100 (1 M KOH)	100 h	Sun et al. (2021b)
MOF MnV oxide	310	—	51.4	--	—	50 (1 M KOH)	100 h	Ji et al. (2023)
MOF CoFeP	310	200	50	42	1.78	50 (1 M KOH)	30 h	Muthurasu et al. (2020)
Co–Fe-MOF	310	—	53	—	1.59	10 (1 M KOH)	10 h	Singh et al. (2024)

In order to overcome to overcome the intrinsic low conductivity of the pristine frameworks, the majority of the systems are designed as MOF-derived or the hybrid architectures (e.g., MOF/NF, MOF@CNT, or MOF-derived carbon composites) as per the detailed assessment of the MOF-based electrocatalysts presented in Table 2. These modified systems exhibited improved electrocatalytic activity with overpotentials typically in the range of ∼230–350 mV for OER, while significantly lower values for HER in the optimized configurations. Interestingly, several catalysts achieve high current densities up to 1,000–2,000 mA cm^−2^ reflecting their potential for the industrial-scale operation, however the performance is often accompanied by the increased overpotentials or limited stability. The reported stability varies greatly in terms of the durability from about 10 h to 100 h, indicating that the structural robustness is still a significant challenge, especially when operating at the high current densities. Additionally, the majority of the systems are assessed in the alkaline media (standard 1 M KOH) emphasizing their relevance to the ALK technologies, while the limited acid-condition studies limits their applicability in PEM system. Notably, enhanced catalytic performance is consistently associated with the MOF-derived conductive substrates and the synergistic metal centers which optimize the adsorption of the reaction intermediates and promote faster charge kinetics. Even though MOFs have shown a wide range of application possibilities there is still a great deal of room for improving their chemical and structural characteristics to improve their performance in electrochemical energy storage and catalysis under extended operations. Fortunately, a novel approach to enhancing the physicochemical features of organic-inorganic materials has been made possible by the extension of the high-entropy technique from inorganic materials (Xing et al., 2025).

### 2.4 High entropy materials

**FIGURE 5:**
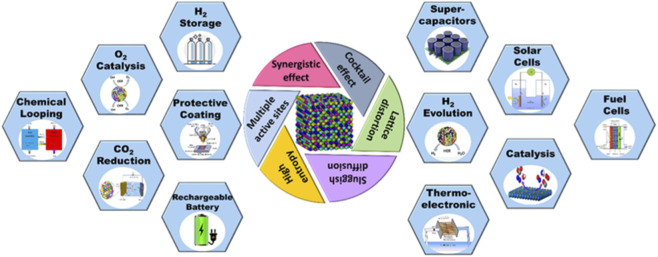
Unique properties of HEMs and their applications in energy storage and conversion technologies.

Due to their special qualities, such as low atomic diffusion, robust structure, a wide range of adsorption energies, and multi-component synergy, high entropy materials (HEMs) with five or more uniformly distributed metal constituents, have become an exciting new category of electrocatalysts. These characteristics render them promising catalysts for complicated electrochemical processes, such as those involving water splitting (Esquius and Liu, 2023). High-entropy materials (HEMs) have unique properties such as slow diffusion and cocktail effects, distinguishing them from conventional materials and making them highly promising (Li C. et al., 2024) electrocatalysts as demonstrated in Figure 5. The architecture of HEMs is highly disordered, with multiple components distributed randomly due to their extremely high configurational entropy (Buckingham et al., 2023). This highly disordered structure benefits from causing a defect-rich structure and uniformly unsaturated coordination sites, which increases the number of readily available active sites. Furthermore, a distributed configuration of different components will produce a high level of homogeneity, which will improve the interaction between different atoms to change their electronic configuration and the synergistic effect, ultimately increasing the intrinsic functionality of each active site (Zhang et al., 2021). Additionally, the special entropy stabilization effect during electrochemical water splitting can make HEMs much more electrochemically stable (Sha et al., 2024).

Based on these beneficial qualities, several HEMs, such as alloys (HEAs), oxides (HEOs), layered hydroxides (HELHs), metal-organic frameworks (HE-MOFs), phosphides (HEMPs), sulfides (HEMSs), and selenides (HESe) have been successfully created and studied. These have been developed as advanced electrocatalysts for OER, HER, and overall water-splitting (OWS) (Ni et al., 2024; Raveendran et al., 2023). Because of their distinct electronically induced effects, entropy stabilization effect, and potent synergistic effect, the prepared HEMs are very active in OER and HER (Wang et al., 2024). Certain elements in HEMs operate as promoters to greatly improve catalytic performance even if they may not directly participate as active sites in water splitting. These promoters affect electron density, oxidation states, adsorption energy (ΔG_H*_), and coordination environment by changing the geometric arrangement and electrical structure of active sites. Improved intrinsic catalytic activity and a synergistic impact result from such alterations, which are essential for maximizing surface characteristics in HEMs. One key mechanism by which these promoters boost their catalytic performance is through electron transfer between promoters and active sites, facilitated by their close interaction (Wu et al., 2025). These characteristics make them potential candidates for electrocatalysts for water splitting. Below is a summary of some of these ground-breaking high entropy materials as sophisticated electrocatalysts for water-splitting:

#### 2.4.1 High entropy MOFs (HE-MOFs)

Blending high-entropy materials and MOFs to build high-entropy MOFs (HE-MOFs) is likely to create new opportunities and exciting capabilities in electrochemical water splitting (Xu X. et al., 2022). High-entropy MOFs are an assortment of solid solutions in a single phase that populate a single sublattice with five or more metal elements. In contrast to popular belief, the high-entropy nature can raise the conformal entropy of the product, stabilizing the single-phase crystal framework (Xia et al., 2024). Five metals (Mo, Zn, Ni, Zn, and Co) have been combined to create a high-entropy metal-organic framework (HE-MOF) through a mild solvothermal technique. Electrical conductivity was improved by this two-dimensional array structure. This electrocatalyst demonstrated outstanding electrocatalytic oxygen evolution reaction (OER) activity and long-term stability for up to 100 h. The coordination and synergy of different metals propel the effective evolution of oxygen (Xu S. et al., 2022).

Mu et al. successfully used a solution-phase technique to create a HE-MOF. Five distinct metal species: Fe^3+^, Co^2+^, Cu^2+^, Mn^2+^, and Ni^2+^ with near-equimolar distribution were distributed at random and coordinated with 1,4-benzene dicarboxylic acid (BDC). The as-synthesised HE-MOF demonstrated exceptional electrocatalytic performance in 1 M KOH towards the OER with an overpotential value of 245 mV, as a result of the strong synergistic effect (Zhao et al., 2019a). Using a solution phase technique, Zhao et al. created a HE-MOF with five components in nearly equimolar concentrations demonstrating strong electrocatalytic activity in an alkaline system. The framework was fabricated by using the solvothermal technique, the Co, Mn, Ni, Cu, and Fe metal species were coordinated with the organic ligands of 2,5-thiophene-dicarboxylic acid and 4,4-bipyridine. When multimetal ions were added, the resulting HE-MOF underwent partial lattice distortion. In contrast to the unaltered MOF with a single metal site, this resulted in the formation of an unsaturated coordination environment surrounding the metal active centers, which increased OER activity with an ultralow overpotential of 310 mV (Yao et al., 2022). Since atomic-scale interactions and thermodynamic/kinetic variables affect catalytic performance, designing next-generation electrocatalysts requires an approach beyond empirical trial-and-error. Understanding the mechanics underlying the OER and HER the use of density functional theory (DFT) is increasingly gaining popularity (Ram et al., 2025). Zeng et al. quite recently leveraged high-throughput DFT and machine learning (ML) to develop entropy-driven design strategies in HITP-based conductive MOFs. The research identifies CoCoZn(HITP)_2_ as the most efficient bifunctional catalyst (total overpotential of 0.39 V) after screening 75 active sites across 35 frameworks. Electronic structure analysis indicates that the alignment of Fe d-states and H s-states is optimal for HER at Fe sites. Additionally, the Co d-band center undergone a downshift, which promotes electron transfer to *OH intermediates and enhance OER activity. Additionally, stacking ensemble ML framework predicts bifunctional activity effectively (*R* ^2^ = 0.907), highlighting electron affinity and valence electron count as key descriptors (Zeng et al., 2026)

#### 2.4.2 High entropy alloys (HEAs)

High-entropy alloys (HEAs) have recently garnered attention in the field of electrochemical water splitting due to their intriguing cocktail effect, wide design space, adaptable electronic structure, and entropy stabilization attributes. Leveraging the complexity and tunability of HEAs allows for a wide range of active sites, maximizing adsorption activity and strength for water splitting through electrocatalytic process (Ahmad et al., 2024). High-entropy alloys (HEAs) are perfect for electrocatalytic processes because of their special qualities, which include exceptional mechanical strength and resistance to corrosion in harsh environments (Tomboc et al., 2020). These properties are a result of slow diffusion as the lattice distortion and the number of components increases. Alloying can dramatically alter the reaction intermediaries’ adsorption energy on the catalyst surface during electrocatalysis, enhancing the electrocatalyst’s catalytic activity (Li et al., 2021).

In comparison to commercial Ni foam, a high-entropy electrode with a hierarchically porous structure (PHEA) was engineered, providing greater mechanical strength, a larger surface area, and optimal electrocatalytic activity. At a high industrial-level current density of roughly 1 A cm^−2^, the bifunctional high entropy electrode showed long-term reliability (Zhou et al., 2024). To determine vanadium’s contribution to enhancing electrocatalytic activity for the hydrogen evolution reaction (HER), Sivanatham et al. reported VxCuCoNiFeMn HEAs. According to structural studies, V has been successfully incorporated, producing an overpotential of 250 mV as well as enhanced electrochemical surface area (ECSA) and electrical conductivity. The fabrication of highly active HEAs for commercial electrochemical water splitting is made possible by this work (Sivanantham et al., 2023). A new catalyst, FeCoNiCuMn high-entropy alloy particles, is being developed to accelerate water splitting. The catalysts outperform the cutting-edge precious metal catalyst RuO_2_ in terms of OER and HER performance, overpotential, and cycle stability of 30 h. The size reduction and lattice distortion of HEAs are responsible for the remarkable catalytic efficiency, underscoring the significance of the intrinsic activity and structure of the catalyst (Yu et al., 2024). Wan et al. prepared a PtFeCoNiCuCr@HCS, much more durable than commercial 20% Pt/C in a KOH environment, with an overpotential of 29 mV at 10 mA cm^−2^ for HER (Wan et al., 2025). In this HEA, the primary active site for water dissociation that produced hydrogen intermediates was Co. These intermediates moved preferentially toward Pt, Fe, and Cu sites, where H_2_ molecules were formed. Additionally, the catalyst surface may experience compressive strain due to the size mismatch between various elements and active sites in HEAs, which increases catalytic activity, promoting the HER process by promoting an optimum H* adsorption.

#### 2.4.3 High entropy oxides (HEOs)

Recent advances in material synthesis have made it possible to create high-entropy oxides (HEOs), which are mainly single-phase metal oxides (MOs) with five or more metal cations on the exact same lattice site (Kante et al., 2023; Sarkar et al., 2019). Unlike high-entropy metal alloys, HEOs allow for targeted construction of the materials’ physical properties because of their additional sublattices. For example, increasing the number of microstates accessible by the macroscopic system can result in rich magnetic properties (Sharma et al., 2020). Additionally, the covalency of the electronic states that are employed and their mixed oxygen transition metal orbital (TMO) character enable additional optimization of the charge transfer characteristics, adsorption energies, (Heymann et al., 2022), and even the availability of an oxygen-related active site for electrocatalysts. As a result, HEOs have already demonstrated great potential in the roles of electrocatalysts and battery electrodes (Bahadur et al., 2018).

A high-entropy composite oxide containing five elements: Co, Fe, Ni, Cr, and Cu was presented by Liu et al. for electrocatalytic water splitting. This high entropy electrode provides a more affordable option for water splitting than CuO because it exhibits quicker charge transfer and better OER performance with an ultralow overpotential and Tafel slope (Liu et al., 2023). In order to create high-activity HEO electrocatalysts, a surface activation strategy that imports plenty of surface oxygen vacancies has been proposed. By demonstrating strong electrocatalytic activity at a low cell voltage of 1.55V and attaining a current density of 10 mA cm^−2^, the modulated HEO opens the door to effective electrocatalytic possibilities (Liao S. et al., 2023). Using a simple coprecipitation technique, high-entropy oxide IrRuCrFeCoNiOx is created, yielding amorphous catalysts with a large specific surface area. With a low Tafel slope and a minimal overpotential, this HEO demonstrated optimal OER catalytic performance. Catalyst stability was enhanced by the formation of a crystalline active layer through surface self-reconstruction. Consequently, a novel OER catalyst for water splitting is developed that exhibits exceptional activity and stability (Rong et al., 2024). Shaikh et al. proposed a high-entropy metal oxide (HEMO)-CeO2 heterostructure that demonstrated noteworthy enhancements in the OER and HER when potassium iodide (KI) was added to the electrolyte (Shaikh et al., 2026). The OER overpotential decreased from 380 mV to 209 mV with KI, and HER overpotential improved from 223 mV to 148 mV, showcasing increased catalytic activity in alkaline conditions. In a bifunctional cell, the voltage requirement dropped from 1.7 V to 1.5 V during prolonged use, indicating that KI-integrated electrolytes can significantly enhance HEMO-CeO_2_ performance for sustainable hydrogen production.

#### 2.4.4 High entropy sulfides (HESs)

High-entropy metal sulfides (HESs) are single-phase solid solutions comprising at least five different types of cations in a sulfide framework, resulting from homogeneously blended multi-metallic compounds (Cui M. et al., 2021). The optimized configurational entropy of HESs over bimetallic or trimetallic sulfides confers remarkable physicochemical and mechanical characteristics, including remarkable stability, outstanding temperature resilience, and optimistic resistance (Mosinoiu et al., 2024). There are currently very few reports on the successful development of high-entropy metal sulfides (Buckingham et al., 2022). By employing a mild cation exchange approach, an intriguing HES (CoZnCdCuMnS@CF) nanoarray has been designed on carbon fiber (CF) support. In alkaline media, this nanoarray shows excellent catalytic efficiency and stability for water splitting for HER and OER, it needs low overpotentials and outstanding durability. After operating continuously for 73 h, the bifunctional electrode for water splitting can generate a small cell voltage and show minimal decay (Lei et al., 2022).

To mitigate the immiscibility of several metallic constituents, glycerol-assisted self-template synthesis was used to create HES (CoFeNiMnCu)S_2_ nanoparticles with a pyrite phase. Through integration into a three-electrode setup, the electronic configuration of the nanoparticles was defined and their exceptional efficacy was further enhanced for OER reaction. These high entropy sulfide nanoparticles showed an exceptionally low overpotential of 284 mV and a Tafel slope of 57 mV/dec (Moradi et al., 2022). Xiao et al. synthesized bulk particulate high entropy ZnS analogs featuring different metals (Cu, Co, Ag, Ga, Mn, Zn, In) using a molecular precursor cocktail strategy. When paired with conducting carbon black, the entropy-stabilized metal sulfide turned out to be a promising electrocatalyst for HER, attaining a low onset potential and η_10_ of about 80 mV (Xiao et al., 2023).

#### 2.4.5 High entropy phosphides (HEPs)

The ligand and strain effects of high-entropy metal phosphides result in greater catalytic activity compared to monometallic or bimetallic phosphides. The ligand effect modifies the electronic structure because different metals’ capacities to donate electrons would cause the valence electrons to be redistributed, creating more active sites and facilitating redox reactions (Zhang N. et al., 2022). Lattice distortions can produce a new electronic arrangement that is advantageous for electrocatalytic HER by altering the local adsorption energy of the ions or reaction intermediates, or by adjusting the d-band center according to the Fermi level through the strain effect (Guo et al., 2023). Due to metal ions and phosphorus incompatibility, there have not been many reports on the engineering of high-entropy metal phosphides for OER. However, Liu et al. have recently engineered an N-doped C high entropy phosphide (HEP), using a simple ZIF-derived phosphide approach and it demonstrated exceptional catalytic behavior with a 199 mV overpotential and 140 h of continuous reaction without compromising efficiency (Liu Z. et al., 2024).

Fast electrodeposition is used to create the Co, Ni, Fe, and Mn-based amorphous high-entropy phosphoxide electrode. On alkaline water/seawater splitting, it demonstrates excellent bifunctional electrocatalytic efficiency, with low HER and OER overpotentials, reaching a 10 mA cm^−2^ current density at low voltages. The amorphous framework, multi-component synergistic effect, advantageous electronic configuration modulation, and surface reconstruction of this material render it a potentially viable alternative to noble-metal electrocatalysts in the water/seawater splitting industry (Zhang H-M. et al., 2024). Zhang et al. introduced CuCrFeNiCoP, a dendritic high-entropy electrocatalyst. Because of its synergistic effect with metal elements, it enhances both hydrogen (HER) and oxygen (OER) evolution reactions performance. The dendrites exhibit exceptional electrochemical stability at a significant current density of 100 mA cm^−2^, making them suitable for energy conversion and preservation. High entropy materials can be synthesized on a large scale with this technique, making it possible to commercialize them for use in energy-related applications (Zhang T. et al., 2023). Zhang et al. recently proposed a novel idea of a high entropy metallic–high entropy nonmetallic community (HEM–HENMC) in order to create an electrochemical catalyst with great efficiency (Zhang C. et al., 2024). They created a HEM–HENMC including five metals (Cr, Mn, Fe, Co, Ni) and five non-metals (C, N, O, P, S) following simultaneous phosphorization, vulcanization, and surface oxidation. The synergistic effect of these ions results in exceptional electrochemical activity. It exhibits a low overpotential of 211.9 mV (@10 mA cm^−2^) and remains stable for more than 25 h as an OER electrocatalyst. This research lays a crucial foundation for developing next-generation high entropy metallic–high entropy nonmetallic materials.

Although the high-entropy systems significantly benefit from synergistic effects and the compositional complexity, it is still challenging to regulate and fully comprehend their catalytic mechanism (Zheng et al., 2026). Additionally, HEMs experience sluggish diffusion, which negatively affects kinetics and phase transitions. The distortion in the lattice caused by multiple elements with different atomic radii and chemical properties hinders atomic movement and reduces diffusion rates. This suppressed diffusion not only affects phase stability but also critically influences the overall performance of the HEM system. Future studies could solve these challenges by integrating high-valence metals (like Mo, W), stabilizing elements, and fabrication of the spinel structure (that leads to the formation of spinel oxide surface layer during the electrochemical reaction) which gives the electrocatalysts self-healing capabilities that allow them to sustain long-term stability and high HER/OER activity protection against corrosion and dissolution under challenging electrochemical circumstances (Zhao et al., 2025). Furthermore, non-metals (such as N, C, O, P, F, S, B, *etc.*) can act also be employed as promoters to enhance the OER process. These species enhance the orbital overlap between the multiple metal centers and their neighboring anionic ligands, thereby improving the covalency of metal-anion bonds. This modulation facilitates the efficient charge redistribution across the disordered lattice, thus leading to the stabilized high-valence metal cations that lower the energy barriers for the intermediate adsorption and accelerate OER kinetics (Zhu J. et al., 2026).

In contrast perovskites’ ease of synthesis, low cost, well-defined crystal structures, improved stability, strong scalability, and intriguing catalytic characteristics have attracted a lot of scientific attention. Metal-oxygen bonds in perovskites deliver high-energy surface sites with asymmetrical coordination number, leading to the unique surface properties and configurable electronic structure (Iqra et al., 2026).

### 2.5 Perovskite oxides

**FIGURE 6:**
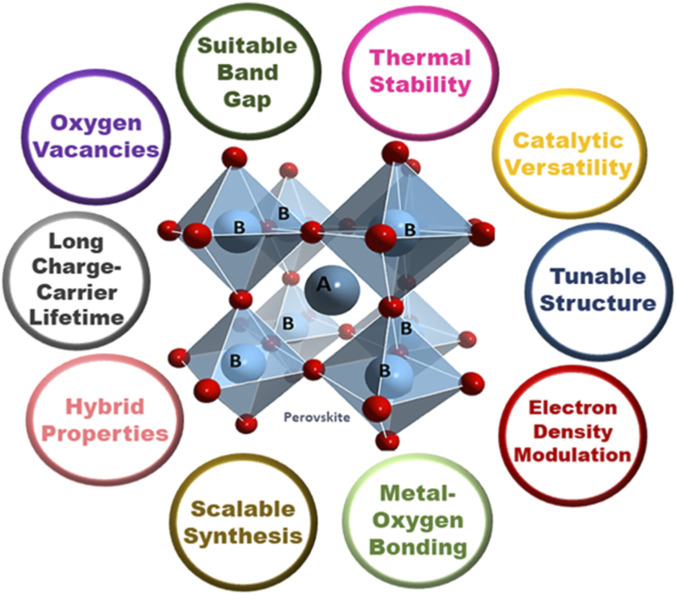
Properties of perovskites enabling high catalytic activity.

Recently, there has been an interest in investigating more effective OER and HER electrocatalysts for the electrochemical water splitting. Perovskite oxides surpass the noble metals IrO_2_ and RuO_2_ in intrinsic activity, and among the many candidates, they exhibit excellent catalytic activity for OER, HER, and fuel oxidation (Beall et al., 2021). Perovskite-type oxides have attracted significant consideration recently due to their excellent electronic conductivity, exceptionally high catalytic activity, and solid crystal structure (Kaleeswarran et al., 2021). Since perovskite oxides have tunable compositions and structures that provide many possibilities and approaches for designing desired electrocatalysts, they have been widely used as effective and affordable catalysts in many fields (Sun et al., 2020). Numerous studies have examined their structural benefits and controllable physico-chemical characteristics in energy storage applications (Mohan et al., 2023). It has the special advantages of being inexpensive, simple to synthesize, and having a controllable electronic structure when compared to other transition metal oxide electrocatalysts (Lyu et al., 2023). The properties of perovskites as electrocatalytically proficient materials are represented in Figure 6.

A common structure found in perovskites is represented by the symbol ABO_3-δ_, in which the cations 'A' and 'B' are of different sizes (Dawa and Sajjadi, 2024), and the anion 'O' forms bonds with both cations. Where A is usually an alkali/alkaline or rare earth metal and B is a transition metal (Si et al., 2022). Interestingly, almost 90% of the elements in the periodic table can be integrated into the A/B-site or O-site of the perovskite structure, which would control the electrical properties of the resulting perovskite oxide. Through the substitution of the A- and the B- sites, the electronic structure of the perovskite can be tuned, which directly modulates e_.g.,_ orbital occupancy and d-band center relative to the Fermi level, thereby improving metal-oxygen hybridization and covalency. This lowers the energy needed to create an O vacancy and encourages ionic conduction in perovskites, a feature that serves as the foundation for its use in electrocatalysis (Bora et al., 2025). This flexibility the Furthermore, a wide range of oxygen non-stoichiometry (δ) values is possible, opening up numerous options for modifying the electronic structure and enhancing charge transfer (Wang et al., 2023). The two primary determinants of electrocatalytic performance are intrinsic activity and the number of active sites. Numerous studies have demonstrated that perovskite’s morphology can successfully control the electrocatalytic activity of HER and OER (Hardin et al., 2014).

Xu et al. developed perovskites with superior HER and OER activity on nickel-iron alloy foam, La_0.9_CoFe/NFF, outperforming many other HER/OER perovskite catalysts. With a 20-h durability, the porous skeleton inhibits aggregation and delayed corrosion and dissolution. This work has prompted an exploration of robust, high-performance dual-functioning perovskite electrocatalysts for overall water splitting (OWS) in large-scale industrial applications (Xu X. et al., 2024). Electrocatalytic efficiency of strontium (Sr), cobalt (Co), and molybdenum (Mo) oxide (SCMO) based on double perovskite in water-splitting reactions, particularly OER and HER, under intense alkaline conditions, was reported by Atif et al. With a significant active surface area of 26.3 cm^2^ and consistent performance over 24 h in harsh alkaline media, the perovskite oxide, created *via* a scalable auto-combustion approach, demonstrated outstanding efficiency (Atif et al., 2024). A straightforward method based on MoS_2_ and its hybridization with LaCoO_3_ perovskite oxide has been designed to adjust the intrinsic characteristics of a TMD. Due to intriguing flower-like morphology, perovskite hybridization enhanced water splitting with a low Tafel slope and onset potential and increased surface-active edge sites. Furthermore, at a notable current density of 50 mA cm^–2^, the system showed sustained stability during 400 h of continuous operation (Rana et al., 2022). These findings could lead to the development of bifunctional catalysts for large-scale overall water splitting that are more straightforward and less expensive. Liu et al. synthesized a LaCo_0.67_Cu_0.33_O_3_ (LCCO) composite perovskite electrocatalyst using a sol–gel method and acid etching for defect engineering (LCCO-x, where x = 6, 12, 24, and 30, indicating treatment time in hours) (Liu et al., 2025). LCCO-24 demonstrated high activity and enhanced reaction kinetics for both the OER and HER in alkaline conditions (1M KOH), achieving a low full-cell voltage of 1.49 V at a current density of 10 mA cm^–2^ during overall water splitting with a stability for upto 25 h, comparable to leading noble metal catalysts. The increased bifunctional electrocatalytic activity was linked to a higher presence of oxygen defects and greater surface area. Table 3 depicts the electrocatalytic efficiencies of perovskites for water-splitting in alkaline media.

**TABLE 3:** Analysis of the performance of perovskite-based electrocatalysts in alkaline media.

Catalyst	Electrolyte media	System	Overall cell voltage(V)	Current density	Durability	References
SrNb_0.1_Co_0.7_Fe_0.2_O_3-δ_ (SNCF) NRs	0.1 M KOH	Alkaline	1.68	10 mAcm^−2^	30 h	Zhu et al. (2017)
RP/SP	6 M KOH	AEM	2.00	2.01 Acm^−2^	48 h	Tang et al. (2022)
SrIrO_3_	1 M KOH	Alkaline	1.59	10 mAcm^−2^	10 h	Yu et al. (2020b)
Pr_0.7_Sr_0_ . _3_Co_1_ – *_x_* Ru*_x_* O_3_ (PSCR0.05)	1 M KOH	Alkaline	1.77	10 mAcm^−2^	10 h	Zhao et al. (2023)
SrFe_0.7_Ru_0.3_O_3-δ_ (SFR30)	1 M KOH	Alkaline	1.58	10 mAcm^−2^	96 h	Zhang et al. (2024d)
SrCo_0.5_Fe_0.4_Mo_0.1_O_3-δ_ (SCFM10)	1 M KOH	Alkaline	1.68	10 mAcm^−2^	100 h	Zhang et al. (2024e)
PrSr_3_Fe_1.5_Co_1.5_O_10−*δ*_ (PSFC)	0.1 M KOH	Alkaline	1.628	20 mAcm^−2^	90 h	Zhu et al. (2023)
Nd_2_CoIrO_6_	0.5 M H2SO4	Acidic	1.53	10 mAcm^−2^	48 h	Ganguly et al. (2023)
EBSCF0.4–20RuO_2_	1 M KOH	Alkaline	1.47	10 mAcm^−2^	24 h	Wang et al. (2020d)
La_1.4_Sr_0.6_NiMnO_6_	1 M KOH	Alkaline	0.86	1.0 mAcm^−2^	24 h	Qu et al. (2021)
Sr_2_RuO_4_ (SRO)	0.5 M H2SO4	Acidic	—	1,000 mAcm^−2^	56 days	Zhang et al. (2022b)
	1 M KOH	Alkaline			∼30 days	
BiCoO_3_	1 M KOH	Alkaline	—	1,000 mAcm^−2^	100 h	Hu et al. (2023)
LSC&MoSe_2_	1 M KOH	Alkaline	2.16	100 mAcm^−2^	1,000 h	Oh et al. (2019)
PB_0.94_C-DSPH	1 M KOH	Alkaline	1.93	500 mAcm^−2^	10 h	Liu et al. (2021b)
LaMn_0.5_Ni_0.5_O_3_	1 M NaOH	Alkaline	1.48 (@10 mAcm^−2^)	10, 100 mAcm^−2^	100 h	Hong et al. (2022)

In the context of electrochemical water splitting, studies have demonstrated that defect engineering is a useful method for investigating perovskite oxides. It can be noticed that for the perovskite materials the reported electrochemical performance is evaluated against a gradient of the KOH concentration: 0.1 M (intrinsic activity), 1.0 M (standard), and 6.0 M (accelerated industrial stress), establishing comprehensive profile of the catalyst kinetics and the material durability. The catalysts, like Nd_2_CoIrO_6_ shows good stability and performance in acidic media, but the use of noble metal Ir limits is adoption due to high-costs for large-scale applications. Upon evaluating the diverse perovskite systems as synthesized in Table 3, a general overarching trend become apparent that the catalytic superiority of such materials is not bound to a single composition. It is rather attributable to the intrinsic density of oxygen vacancies within the lattice. The intentional introduction of the defects acts as a universal performance booster across both of the HER and OER whether through that of the surface etching (as can be observed in the LCCO series) or the B-site doping (as evident in the Nb and Fe-based perovskite systems). The findings suggest that despite the overpotentials may vary, the optimization of the defect chemistry (as can be observed for LCCO-24 and LSC&MoSe_2_ with 1,000 h stability) in order to minimize the ionic transport barriers still remain the fundamental design for the high-performing perovskites. This cohesive strategy explains why a wide variety of the modified perovskites can now compete with that of the commercial noble benchmark materials, offering scalable model for a number of high-efficiency energy applications. Additionally, it was also noted that while the electrocatalytic activity for most of the materials is reaching the commercial levels (500–1,000 mAcm^−2^), the critical evaluation of these trends reflects that the long-term stability remains to be the final obstacle for the industrial adoption. Interestingly, LaMn_0.5_Ni_0.5_O_3_ which is prepared *via* environmentally friendly deep eutectic solvent (DES) was evaluated in the 1 M NaOH to validate its electrocatalytic effectiveness in cost-effective alkaline media despite the low mobility of the Na^+^ compared to K^+^, demonstrating good catalytic efficiency.

Despite their numerous advantages, perovskite materials face degradation from environmental stressors like moisture, oxygen, light exposure, and temperature changes, which can cause permanent structural and chemical damage which hinder their long-term operational durability. This degradation is attributed to intrinsic factors such as weak ionic bonding and halide ion migration, interfacial issues from energy-level mismatches with charge transport layers. These combined effects result in the breakdown of the perovskite lattice, creation of trap states, and significant performance decline over time. To overcome these challenges, 2D materials, particularly the graphene and its derivatives have garnered a lot of attention due to their multifunctional role in enhancing device durability and charge transport (Hasan et al., 2026).

### 2.6 Graphene-based materials

Because of its remarkable properties, such as its massive theoretical surface area (nearly ≈2.63 × 103 m^2^ g^−1^), outstanding electrical conduction (roughly 5000 W m^−1^ K^−1^), chemical stability, mechanical strength (Young’s modulus, ≈1.0 TPa), honeycomb lattice, high optical transparency (≈97.7%), defect engineering, catalyst support, exceptional charge carrier mobility (2 × 10^5^ cm^2^ V^−1^ s^−1^), easy tunability, and more, graphene and graphene-based materials have recently offered an intriguing platform for the engineering of effective electrocatalyst materials (Sutar and Yadav, 2024) (Tareen et al., 2022). Its electrocatalytic performances have been significantly improved through hybrid fabrication, surface and defect engineering, and metal and non-metal doping (Nemiwal et al., 2021b). The remarkable optoelectronic and physicochemical attributes of graphene-based derivatives, combined with their distinct layered structure, effectively mitigate the thermodynamic uphill effect of water splitting compared to their non-layered counterparts (Sadiq et al., 2023). Many graphene-based materials have shown promise as electrocatalysts, such as reduced graphene oxide (rGO), graphene quantum dots (GQD), and single, double, and ternary-doped graphene (Akhter et al., 2024). In graphene-based systems, the catalytic performance is primarily governed by the heteroatom doping (N, P, S, *etc.*), defect engineering and synergistic interactions with the incorporated species. These modifications induces charge redistribution in that of the sp^2^-conjugated graphene lattice, generating the catalytically active sites and optimizing the adsorption energy of the key intermediate species (ΔG_H*=_≈ 0 for HER and *OH, *O, *OOH for OER) (Dutta et al., 2025). Furthermore, upon hybridization with the metal species graphene acts as a conductive scaffold that promotes the uniform dispersion of active sites and prevent particle agglomeration, and enhance charge transfer, ultimately improving the catalytic activity for both HER and OER (Nazir et al., 2025).

Recently a NiS/rGO nanocomposite has been developed by a hydrothermal process, which is favorable for commercial applications owing to its greater catalytic efficiency, affordability, and accessibility. The composite is stable for about 40 h due to its unique structure and more active regions. Increased conductivity from adding carbon-based reduced graphene oxide results in a larger surface area of about 2852.5 cm^2^, minimal overpotential (162 mV), and a lower Rct value (0.07 Ω). Following this study, rGO paired with metal sulfide might be an affordable option for OER applications in the future (Majeed et al., 2024). Composites of graphene quantum dots (GQDs) and ZSM-5 type cobaltosilicate have been developed for electrochemical water splitting, which requires lower charge transfer resistance, Warburg impedance, and overpotentials. This could lead to new developments in materials science and electrocatalytic processes (Mokhtari et al., 2023). Using a hydrothermal technique, Reghunath et al. created a ternary composite consisting of N-doped graphene quantum dots, graphitic carbon nitride, and cobalt ferrite. The Tafel slopes for HER and OER were 94 mV dec^−1^ and 69 mV dec^−1^, respectively, indicating low overpotential for overall water splitting. To achieve a current density of 10 mA cm^−2^, the electrode required a cell potential of just 2.0 V in the overall water splitting (Reghunath et al., 2023). To improve electrocatalytic performance, Noreen et al. created La-Ni based MOFs in conjunction with graphene oxide (GO) (Noreen et al., 2026). With Tafel slopes of 51 mV dec^−1^ for HER and 47 mV dec^−1^ for OER, the La-Ni-MOF/GO material showed dual functionality, producing hydrogen at 0.39 V and oxygen at 2.31 V in 1 M KOH (pH = 13.6). With a high turnover frequency of TOF = 1.34 s^−1^ and low charge transfer resistance (Rct = 4.3 Ω for OER and 556 Ω for HER), it demonstrated exceptional stability over 50 h. This research demonstrated the potential of integrating bimetallic MOFs with GO to produce novel bifunctional electrocatalytic materials with improved charge transfer, more accessible active sites, and long-term stability. In order to promote future advancements in renewable energy conversion technologies, this innovative low-cost scalable approach will offer an incredibly high-performance durable catalyst for H_2_ generation. Table 4 lists a few of the most effective graphene electrocatalysts.

**TABLE 4:** Overview of the electrochemical behavior of graphene-based materials for ECWS.

Catalyst	Electrolyte medium	Overpotential (mV)		Overall voltage (V) @10 (mAcm^−2^)	Tafel slope (mVdec^−1^)		Durability	References
		OER	HER		OER	HER		
NiCoS@CNT/Graphene	1.0 M KOH	295	198	1.53	78	87	38 h	Ashok et al. (2020)
CuNi@Ni(ON)/CNTs-Gr	1.0 M KOH	410	42.1	1.51 V	257	60	25 h	Tran et al. (2021)
RuO_2_-P-rGO	0.5 M H_2_SO_4_	194	43	1.42	94	28	58 min	Raja et al. (2022)
MoC/NiC@N-rGO	1.0 M KOH	298	185	—	80	78	55 h	Al et al. (2024)
rGO/Ni-Foam	1.0 M KOH	400	169	—	104	108.23	—	Kumar et al. (2023)
NiSe2/rGO-ST	1.0 M KOH	235	52	1.75	236 (@30 mAcm^−2^)	67	12 h	Amin et al. (2023)
CoP/rGO-400	1.0 M KOH	340	150	1.70	66	38	22 h	Jiao et al. (2016)
0.8 GO-FeNiLDH	1.0 M KOH	285	119	1.48	33	36	25 h	Han et al. (2019)
CuWO_4_@rGO	0.5 M H_2_SO_4_	∼330	∼135	—	∼315	∼212	10,000 s	Ahmed et al. (2022)
CuWO_4_@rGO	0.5 M KOH	∼270	∼180	—	∼315	∼192	10,000 s	
GH-BGQD2	0.1 M KOH	370	130	1.61	70	95	70 h	Tam et al. (2019)
Ni_3_S_2_-NGQDs/NF	1.0 M KOH	216	218	1.58	95.5	89.0	16.6 h	Lv et al. (2017)
G-Mo-Ni_3_S_2_	1.0 M KOH	326 mV (@20 mA cm^−2^)	68	1.58	69	30	50h	Li et al. (2019)
Ir@N-G-600	0.5 M H_2_SO_4_	314.6	43.9 (@ 30 mAcm^−2^)	1.579	74	27	27 h	Yi et al. (2021)
Ir@N-G-600	1.0 M KOH	291.8	92.5	1.566	45	41	27 h	
Ru/BN@C	0.5 M H_2_SO_4_	—	35	—	—	37.66	50 h	Salah et al. (2023)
	1.0 M KOH	—	32	—	—	33.89	50 h	
Ni@MnS/SGCN nanocomposite	1.0 M KOH	380	650	—	34.65	50.16	18 h	Bashir et al. (2025)

A critical analysis of the graphene-based materials listed in Table 4 reveals that the catalytic performance is strongly governed by that of the compositional design and the operating environment. Although majority of the materials are tailored for either acidic or basic media, a subset of materials demonstrates pH-universal activity in both operating conditions. Apart from the noble-integrated systems like the Ir@N-G-600, non-noble metal graphene hybrids like CuWO_4_@rGO exhibit competitive bifunctional activity (HER/OER) in both of the acidic (H_2_SO_4_) and alkaline (KOH) media. This indicates that the appropriate hetero-atom doping, defect engineering and the metal-support interaction can facilitate the catalytic behavior that is independent of the reaction media. However, stability remains to be the key differentiator from the structural and mechanistic standpoint. Additionally, the corresponding high Tafel slope values indicate the low electrocatalytic efficiency. Under the acidic conditions pertinent to the PEM systems, metal leaching and corrosion resistance is extremely important (Feng et al., 2025). This explain the continued reliance of the high-performing electrocatalysts on that of the noble metals, which hinders their large-scale, cost-effective adoption (Zhang B. et al., 2026). On the other hand, graphene materials based on the non-noble metals (such as Ni, Fe, Mo, Co, or Cu-modified systems has the potential to deliver high activity through improved charge kinetics and the optimized adsorption energetics in the alkaline environment which are relevant to the AEM and the traditional alkaline elctrolyzer. However, for scalability and the industrial applications, absence of the performance at high-current densities highlights the need for additional advancements.

The development of pH-universal non-noble catalysts represent a shift towards the corrosion-resistant materials that can bridge the gap between those of the PEM and the AEM technologies. These findings suggest that the catalytic performance is greatly impacted by the structural stability and the environmental compatibility rather than being solely material dependent. However, the scarcity of material that are operational in dual-environment and challenges like the limited durability in the acidic media and graphene restacking, emphasize the necessity of creating the robust, non-noble graphene-based hybrids that are capable of maintaining catalytic efficiency under a variety of the operating conditions.

Graphene, LDHs, and MOFs serve as substrates for metal single atom loading due to their high specific surface area and strong metal-support interactions. However, using graphene or other conductive agents can lead to active site uncertainty and potential catalytic damage. LDHs and MOFs are also sensitive to acidic environments, limiting their use in energy conversion applications like PEM water electrolyzers. Furthermore, traditional synthesis methods for these materials often result in stable M-N4 or M-OH (M = metal center) in MOFs or LDHs, which restrict their tunability and flexibility. Covalent organic frameworks (COFs) and their derivatives, have recently been extensively reported as electrocatalysts for HER and OER due to their resilience to a broad pH range, earth-abundant, affordability, and mechanical robustness (Zhang Z. et al., 2024).

### 2.7 Covalent organic frameworks (COFs)

Due to their tendency to offer an outstanding platform on which the electrocatalytic active site can settle, COFs have drawn a lot of research interest as promising water-splitting electrocatalysts (Yusran et al., 2023). Since atomic covalent bonds are formed, COFs have greater surface areas, are easier to functionalize, and have more variety than other organic polymers (Dhanabalan et al., 2024). COFs provide an unparalleled array of crucial characteristics, such as a highly ordered structure and a precisely tuned chemical composition, to support the electrochemical processes (Yu C. et al., 2022). Electroactive components can be modularly integrated into structures to provide readily available catalytic sites and improved chemical stability strengthened by covalent bonds (Sun J. et al., 2023). Additionally, it is possible to design COFs with fully or better π-conjugated structures, exhibiting tunable electronic band gaps and semiconducting behavior (Liu et al., 2022). As a desirable electrocatalyst and substitute for noble-metal electrodes, all these appealing qualities hold a lot of promise. Although COF research in electrochemical systems is still in its initial phases, this revolutionary material is additionally being investigated for promoting water-splitting electrocatalysis (Yusran et al., 2020).

COFs are constructed from prefabricated building blocks using a variety of chemical formulations and electroactive functional components to produce rigid and electroactive porous polymers (Akinnawo, 2024). Furthermore, a number of COFs demonstrated some remarkable properties, including long-range periodicity, lower skeletal density, substantial specific surface areas (4,210 m^2^ g^−1^), tunable pore sizes (about 4.7 nm), extraordinary thermal and chemical stabilities (up to 600 °C), and exceptionally high charge mobility (∼8.1 cm^2^ V^−1^s^−1^) (Cui X. et al., 2021). Consequently, COFs offer completely exposed and readily available catalytic active sites, which are essential for raising selectivity, efficiency, and lowering the reaction energy barrier. Moreover, nano-to-mesoporous channels allow for the efficient confinement of single atoms, nanoparticles, nanocluster electrocatalysts, and mass-transfer and electron-transfer pathways (Yasin et al., 2022). Furthermore, the majority of COFs’ π-conjugated structures enhance electronic conductivity, which is easily enhanced by adding heteroatoms, adjusting dopants, and modifying the chemical environment (Cao et al., 2024). Beyond the fundamental reaction pathways, the electronic modulation of the rigid organic framework is largely responsible for the COF’s improved electrocatalytic efficacy. The well-defined pores and ordered structure offers precise coordination environment that optimizes the binding energetic of the reaction intermediates, preventing the over-binding of *H and the oxygenated species *OH, *O, *OOH, which usually restrict bulk catalysts. During the prolonged cycling, this structural confinement prevents the creation of inactive metal clusters by producing high density of the isolated active centers (Wang C. et al., 2025). Additionally, by enabling the rapid electrolyte infiltration and the effective release of the evolved gas bubbles, the intrinsic pore channels promote faster interfacial kinetics. Thus, the COF architecture reduces the overall kinetic barrier, especially during the multi-electron-transfer stage of the OER, by decoupling the active site density from mass transport constraints (Zhang Z. et al., 2026).

More significantly, understanding the structure–activity relationship is essential for comprehending the electrocatalytic mechanism and modulation, and this is what the reticular synthesis of COFs offers (Shah et al., 2023). A novel metal-free 2D COF material, C6-TRZ-TFP, produced by solvothermal polycondensation, demonstrated exceptional HER activity in electrochemical water splitting with an extraordinarily low overpotential of 200 mV as well as outstanding catalytic capability retention after electrocatalysis in 0.5 M aqueous H_2_SO_4_, potentially leading to large-scale hydrogen generation (Ruidas et al., 2021). Wang et al. created hybrid materials for electrochemical applications using COF-grafted graphene aerogel. High OER (η_10_ = 300) and HER (η_10_ = 275) performances have been achieved by this *in situ* step-growth polymerization technique, which makes use of 3D conductive networks and easily accessible active sites (Wang Z. et al., 2022). With planar 2D porphyrin and orthogonal 3D spirobifluorene as π-conjugated units, Chen et al. successfully fabricated a rare 3D COF known as 3D-Por-SP-COF. Porphyrin units effectively chelated metal ions, optimizing benefits for electrocatalytic H_2_ production requiring a small onset potentials of 72 mV to reach 1.0 mA cm^−2^ and low overpotential of 175 mV for HER under alkaline conditions (Chen et al., 2024).

Recently novel N–C@COF core-shell microspheres outperformed pure COF and N–C regarding HER performance in 1M KOH. It also showed outstanding HER durability over 24 h and maintained its catalytic efficiency even after 2000 consecutive electrolytic cycles, making it appropriate for use in large-scale industrial applications. The synthesis approach presents a viable route for the cost-efficient development of electrocatalysts (Xu Z. et al., 2024). Recently, Guan et al. prepared a self-supported TD-COF/NFF electrode by integrating a porphyrin-based covalent organic framework (TD-COF) onto a nickel-iron foam (NFF) scaffold. Because of the decreased charge-transfer resistance at the interface, this assemblage showed enhanced HER/OER activity. The Ni-Fe bimetal increased conductivity and stability by improving active-site energetics and encouraging surface self-reconstruction into active metal (oxy)hydroxides. The electrode maintained 100 mA cm^−2^ for 100 h without degradation in 1 M KOH, achieving 10 mA cm^−2^ at an overpotential of 165 mV for HER and 230 mV for OER. With just 1.66 V needed for 10 mA cm^−2^, an alkaline electrolyzer using TD-COF/NFF demonstrated the possibility of scalable and economical water-splitting electrodes using this interfacial coupling technique (Guan et al., 2026). Analysis of the catalytic efficiency of COF-based electrocatalysts for water splitting is given in Table 5.

**TABLE 5:** Evaluation of the electrocatalytic performance of covalent organic frameworks in terms of overpotential, Tafel slope, electrochemical surface area, durability, current density, and double layer capacitance (C_dl_).

Catalyst	Overpotential (mV)	Tafel slope (mV dec^−1^)	Current density (mA cm^−2^)	Double layer capacitance (F cm^−2^)	Electrochemical surface area (cm^2^)	Stability	References
IISERP-COF2	258 (OER)	38.9	10 (0.1 M KOH)	3.98 × 10^−4^	4.7	13 h	Mullangi et al. (2016)
Co-TpBpy	400 (OER)	59	1 (0.1 M PBS, pH 7)	1.286 × 10^−5^	0.286	24 h	Aiyappa et al. (2016)
TpBpy-Co-40000s	380 (OER) (@ 10 mA cm^−2^), 430 (@ 50 mA cm^−2^)	54	10, 50 (0.1 M KOH)	1.93 × 10^−5^	∼0.48	10h (@ 10 mA cm^−2^)	Zhao et al. (2019b)
C4-SHz COF	320 (OER)	39	10 (0.1 M KOH)	2.75 × 10^−3^	68.75	11 h	Mondal et al. (2020)
JLNU-302	91 (HER)	103.88	10 (1 M KOH)	33.6 × 10^−3^	627	20 h	Ma et al. (2022)
c-CNT-0.68@TpBpy-Ru	112 (HER)	160	10 (1 M KOH)	16.2 × 10^−3^	405	12 h	Sun et al. (2024)
CoNPs@JUC−625	146 (HER)	186	10 (1 M KOH)	84 × 10^−3^	—	24 h	Song et al. (2022)
N-MoS_2_/COF-C_4_N (1:1)	106 (HER) 349 (OER)	64 (OER)	10 (0.5 H_2_SO_4_)	8.2 × 10^−3^ (HER) 4.19 × 10^−3^ (OER)	—	20 h	Zhang et al. (2023e)
NGA-COF@Pt	13 (HER)	21.88	10 (0.5 H_2_SO_4_)	4.11 × 10^−3^	102.8	15 h	Zhang et al. (2024f)
MWCNTs@TpPa-COF@Co/CoO	28.8 (HER)	69.07	10 (1 M KOH)	117.25 × 10^−3^	—	24 h	Yang et al. (2024)
IrRu DAS/AT-COF	39.3 (HER), 251.2 (OER)	96.34 (HER), 67.8 (OER)	10 (1 M KOH)	17.23 × 10^−3^	—-	100 h (@ 100 mA cm^−2^)	Ran et al. (2026)

From the assessment of the COF-based electrocatalysts listed in Table 5, it is apparent that the electrocatalytic performance is significantly governed by the framework design, heteroatom incorporation and the π-conjugated electronic structures (Nakatani et al., 2025). Interestingly, Co-TpBpy demonstrated moderate catalytic efficiency in neutral condition demonstrating the ability of COFs to be employed in variety of operating conditions. Unlike the other discussed materials most of the COFs are evaluated at the moderate current densities (10 mA cm^−2^) reflecting their current limitation to the lab-scale applications. It can be observed that reported overpotentials demonstrated significant variations. For the OER the overpotential values typically ranging from ∼250 mV to 400 mV, whereas some of the engineered systems exhibit low HER overpotentials (as low as 13 mV in acidic media), thus highlighting the effectiveness of the electronic modulation and the active site engineering. Notably the parameters like ESCA and the double-layer capacitance demonstrates that the enhanced catalytic efficiency is closely associated to that of the density of the accessible active sites and enhanced charge transfer properties. Even though long-term robustness remains a concern, stability values ranging from ∼10–100 h suggest a considerable durability. Mechanistically the doping with the heteroatom introduces catalytically active sites that maximizes the adsorption of the reaction intermediates, thereby influencing the HER and OER kinetics. Whereas, the π-conjugated frameworks enhance the charge delocalization. However, scarcity of achieving high-current density performance and the research conducted in alkaline settings underscore the need for further development towards the scalable and practical electrocatalytic systems, particularly in a variety of the operating conditions.

Moreover, since COFs are mostly composed of light elements, their physicochemical features, such as electrical conductivity and catalytic activity, are limited. To overcome these limitations, innovative strategies such as integrating COFs with suitable supports or substrates to create composite catalytic architectures have emerged as a critical approach for enhancing their functional applications and addressing intrinsic issues in COF design and synthesis.

**FIGURE 7:**
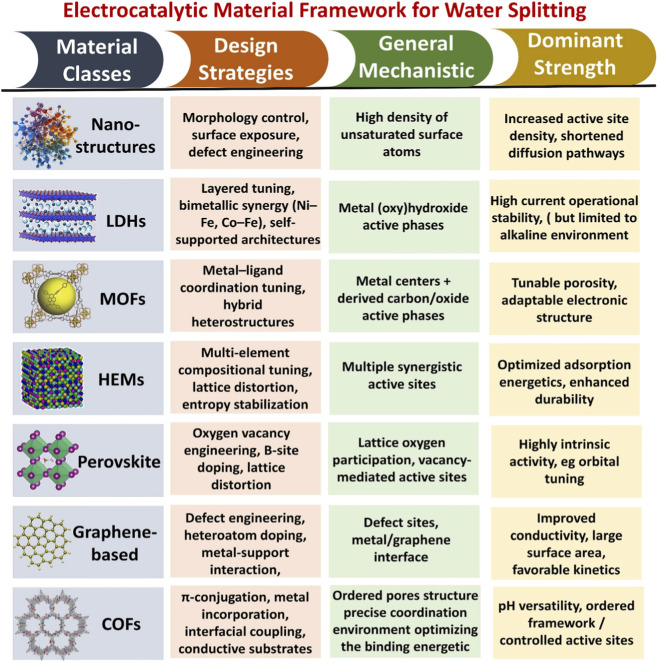
General framework correlating the material classes, design strategies, key mechanistic, and the dominant strength governing the electrocatalytic water splitting.

In order to offer unified perspective across the diverse catalyst systems discussed, a framework based summary is presented in Figure 7. This schematic illustration correlates the diverse catalysts classes with their dominant design strategies, representing active sites, and the generalized mechanistic features. This approach highlights how the different materials systems converge towards that of the common catalytic principles, while also emphasizing their distinguishing strengths.

## 3 Future prospects

Despite the significant advancements, a number of the critical challenges must be addressed to enable the practical deployment of the electrocatalyst for large-scale water splitting. A major limitation lies in achieving the stable operations at industrially relevant current densities (>500 mA cm^−2^) while retaining low overpotentials. Despite the exceptionally high-current-density stability of the LDH-based systems, their performance is largely restricted to that of the alkaline environments. This emphasizes the urgent need for the pH-universal catalysts that are capable of stable operation in both of the acidic and alkaline solutions for sustainable hydrogen production. The performance analysis across the various materials classes further suggested that interface engineering and the hybrid system design represent key directions for the future research and development (Chauhan et al., 2025). Conductive, self-supported architectures such as metal foam-based systems due to their low-density, high porosity and high volumetric surface area, increase the active site accessibility, promote efficient charge transfer and improve structural robustness under the extended electrocatalytic cycles (Novais et al., 2025). Integrating such platforms with the functional materials (such as MOFs, COFs, LDHs, graphene, *etc.*) offers an intriguing opportunity to combine their complementary characteristics including the customizable electronic structures and enhance catalytic kinetics.

Additionally, the dynamic evolution of the catalyst surfaces during the electrocatalytic operations remains insufficiently understood. Many of the pre-catalysts undergo undergoes irreversible surface reconstruction into the amorphous (oxy)hydroxides under the high anodic potentials (particularly in OER) that serve as the active species. Adopting the *in situ*/operando characterization techniques (such as XAS, Raman, TEM, *etc.*) would enable real active site confirmation and observation of structural degradation in real-time (Nguyen et al., 2026). For the rational design of the efficient electrocatalysts, it is critical to identify whether the catalytic activity stems from the original crystalline lattice or reconstructed surface layer. Moreover, computational approaches are increasingly playing an important role in accelerating the design of the electrocatalysts. DFT offers a powerful framework for assessing catalyst performance through quantifying the key descriptors such as Gibbs free energy of the reaction intermediates and electronic properties. However, its computational expense poses challenges in exploring extensive chemical and structural design spaces. Deep learning models have been proposed to facilitate faster predictions of HER/OER activity using DFT-derived datasets (Thomas, 2025). The integration of these methods would enable the development of the predictive structure-activity relationships, thereby guiding the informed engineering of the high-performance electrocatalysts. Collectively, these directions emphasize the transition from the isolated material optimization to that of the integrated system-level electrocatalysts design for the scalable hydrogen production.

## 4 Conclusion

In conclusion, this review offers comprehensive insights and the critical evaluation of advanced materials for the electrochemical water splitting by incorporating material design, mechanistic understanding, and performance evaluation within a unified framework. The findings indicate that the catalytic efficiency is not governed by single material class, but by the interplay between the electronic structure, active site engineering, and system-level architecture. A comparative assessment of various catalyst systems reveals that each material class offers unique benefits ranging from the active site accessibility and stability to the improved charge transport and tunable electronic structures. Nanostructures increase the number of the readily available active sites. LDH-based systems demonstrated high current density in alkaline environments owing to their advantageous redox chemistry and structural adaptability. HEMs enhance catalytic stability through lattice distortions and the multielement interactions. Graphene-based hybrids benefit from defect engineering and electronic modulation in facilitating the efficient charge transfer. Perovskite materials emphasize the significance of creating oxygen vacancies in enhancing catalysis, irrespective of their specific composition. Additionally, MOFs and COFs-derived systems enable precise control over the structural and electronic characteristics allowing for the refinement of active sites at molecular level. These findings highlight the necessity of integrating complementary capabilities rather than depending solely on isolated material systems for the rational design of highly efficient electrocatalysts. Despite significant progress, challenges related to the long-term stability, high-current density and performance under the practical operating condition hinder large-scale implementation. Advancements in the material design, emerging characterization techniques, and computational approaches are expected to play vital role in bridging the gap between the fundamental research and scalable hydrogen production technologies.
